# Acupuncture as an Independent or Adjuvant Management to Standard Care for Perimenopausal Depression: A Systematic Review and Meta-Analysis

**DOI:** 10.3389/fpsyt.2021.666988

**Published:** 2021-05-28

**Authors:** Fei-Yi Zhao, Qiang-Qiang Fu, Gerard A. Kennedy, Russell Conduit, Wen-Jing Zhang, Zhen Zheng

**Affiliations:** ^1^School of Health and Biomedical Sciences, RMIT University, Bundoora, VIC, Australia; ^2^Shanghai Municipal Hospital of Traditional Chinese Medicine, Shanghai University of Traditional Chinese Medicine, Shanghai, China; ^3^Department of Nursing, School of International Medical Technology, Shanghai Sanda University, Shanghai, China; ^4^Yangpu Hospital, Tongji University School of Medicine, Shanghai, China; ^5^School of Science, Psychology and Sport, Federation University, Mount Helen, VIC, Australia; ^6^Institute for Breathing and Sleep, Austin Health, Heidelberg, VIC, Australia

**Keywords:** acupuncture, perimenopausal depression, standard care, systematic review, meta-analysis

## Abstract

**Background:** Many women with perimenopausal depression (PMD) have sought alternative therapies such as acupuncture because of concerns about risks associated with antidepressant and hormone replacement therapy (HRT). This systematic review aimed to clarify if acupuncture is effective for PMD compared with waitlist control or placebo/sham acupuncture, and if acupuncture alone or combined with standard care (antidepressant and/or HRT) is more effective in ameliorating PMD in comparison with standard care alone.

**Methods:** Randomized controlled trials (RCTs) of PMD treatment *via* acupuncture vs. waitlist control or placebo/sham acupuncture, and RCTs of PMD treatment *via* acupuncture alone or combined with Western pharmacotherapy vs. Western pharmacotherapy were searched for from seven databases from inception to December 2020. Cochrane criteria were followed.

**Results:** Twenty-five studies involving 2,213 women were analyzed. Meta-analyses indicated that acupuncture significantly reduced the global scores of Hamilton Depression Scale (HAMD) [standardized mean difference (SMD) = −0.54, 95% CI (−0.91, −0.16), *p* < 0.01], compared with standard care. The therapeutic effect of acupuncture maintained at 2-, 4-, and 12-week follow-ups. Acupuncture combined with standard care was more effective than standard care alone in decreasing HAMD scores [SMD = −0.82, 95% CI (−1.07, −0.58), *p* < 0.01]. Too few RCTs were available to assess the clinical efficacy differences between acupuncture and placebo/sham acupuncture or HRT alone. Acupuncture also showed better effects in decreasing Kupperman index (KI) scores, whether compared with antidepressant alone [MD = −4.55, 95% CI (−8.46, −0.65), *p* = 0.02] or antidepressant combined with HRT [MD = −0.89, 95% CI (−1.34, −0.43), *p* < 0.01].

**Conclusions:** In comparison with standard care, acupuncture alone or combined with standard care was associated with significant improvements in PMD and reductions of other menopausal symptoms. This finding suggests that acupuncture may be a useful addition to treatment for PMD.

## Background

Women are twice as likely to suffer from depression in their lifetime as men, and depression is also one of the major causes of disease-related disabilities in women (1). Menopausal transition, also called perimenopause, refers to a critical stage of dynamic hormonal flux (2) that occurs at midlife in women and is defined as a specific period in the final years of reproductive life (3–5). Experiencing a range of significant endocrine and other biological changes (6), women are usually affected by a variety of physical and psychological complaints, including vasomotor symptoms (hot flashes and night sweats); sleep disturbance; vaginal, urinary, and sexual symptoms (e.g., vaginal dryness, dyspareunia, bleeding, etc.), as well as adverse mood states (e.g., depression, anxiety, mood swings, etc.) (1, 5, 6). Perimenopause is defined as a “window” of vulnerability for the development of depression (7), with prevalence rates of depression ranging up to 20–40% (8, 9). A number of cross-sectional investigations have shown that in comparison with premenopause, women in perimenopause are at a higher risk for depression and present a higher prevalence of depressive symptoms (7). Diagnosis and treatment of perimenopausal depression (PMD) is challenging because it commonly co-occurs with other menopausal symptoms (7). PMD is associated with the impaired functional outcomes, decreased social supports, increased complaints of disability, and lower quality of life, which are not widely reported by perimenopausal women without depression (9–11). Untreated PMD can increase medical morbidity after menopause, including risks of cardiovascular disease, diabetes, and osteoporosis (12). Effective management strategies are therefore required to reduce the negative impact of depression in this vulnerable group (10).

Perimenopausal syndrome including mild-to-moderate and non-long-standing PMD symptoms are often managed with hormone replacement therapy (HRT) (3, 5, 6, 13). Despite its positive effect on mood, HRT is linked with the increased risk of ovarian cancer (14), breast cancer (15), and cardiovascular diseases (16). Furthermore, estrogen therapy has not been approved by the Food and Drug Administration (FDA) to treat PMD and/or other mood disturbances (7). Antidepressant treatment [e.g., selective serotonin reuptake inhibitors (SSRIs), serotonin–norepinephrine reuptake inhibitors (SNRIs), and mirtazapine, etc.] is another pharmacological option for PMD (7). However, women tend not to use antidepressants due to potential side effects such as weight gain (17), gastrointestinal symptoms (18, 19), and sexual dysfunction (17–19).

The limitations of conventional therapies have driven women to seek relief from complementary and alternative medicine (CAM) treatments (20). Acupuncture as part of Traditional Chinese Medicine (TCM) is one of the most popular and safest CAM therapies (21). It is a traditional healing technique involving the insertion of fine, solid, metallic needles into targeted sites called “acupoints” on the body wall to achieve therapeutic outcomes (22–24). After insertion, the needles are usually stimulated manually with slight twisting and with gentle movements up and down [manual acupuncture (MA)] or are stimulated with the electrical impulses delivered by an electric microcurrent device [electroacupuncture (EA)] (22, 24, 25).

Several randomized controlled trials (RCTs) regarding the use acupuncture for the treatment of PMD have been published (26–29). Conflicting findings (30, 31) and differences in research design among those RCTs hinder a firm conclusion regarding the use of acupuncture for PMD (27), as either an independent or adjuvant therapy to standard care (antidepressant and/or HRT). This systematic review aimed to address the following research questions: (1) Can acupuncture be used as an independent therapy for PMD?; (2) how effective is acupuncture for the management of PMD in comparison with standard care; and (3) when acupuncture is used as an adjuvant therapy to standard care, could it further enhance the therapeutic effect or reduce the side effects of Western pharmacotherapy? This systematic review was carried out in accordance with Cochrane Handbook for Systematic Reviews and was reported following the Preferred Reporting Items for Systematic Reviews and Meta-Analyses (PRISMA) statement guidelines.

## Materials and Methods

### Study Registration

The protocol for this systematic review was registered in the Prospective Register of Systematic Reviews (PROSPERO): No. CRD42021227015.

### Eligibility Criteria

Studies included were published RCTs with parallel designs. Women in the perimenopausal period with a clinical diagnosis of depression as per standard diagnostic criteria were included. Any trial without a standard diagnostic guideline was excluded even it mentioned that the patient was diagnosed with PMD or it provided brief information regarding women's complaints of depressed mood. Participants in a pre- or post-menopausal status, or with comorbid cardiovascular disease, cerebrovascular disease, endocrine diseases, cancer, other psychiatric or gynecological disorders, or other severe disorders were excluded. Interventions were restricted to traditional needle acupuncture (TNA) including MA and EA, or TNA combined with standard care for PMD (antidepressant and/or HRT). Comparator interventions were restricted to waitlist control, placebo/sham acupuncture, or standard care. The primary outcome was validated depression scales [e.g., Hamilton Depression Scale (HAMD) and Self-rating Depression Scale (SDS)]. There are several versions of HAMD, such as HAMD-6, HAMD-17, HAMD-21, HAMD-23, HAMD-24, and HAMD-27 (32). There was no restriction on HAMD version for searching and including studies. Papers were excluded if they did not report the global scores of any validated depression scale, even though they reported the clinical effectiveness rates based on the scale or reported partial items of the scale. Secondary outcomes included menopausal symptoms assessed with validated scales [e.g., Kupperman index (KI) and Menopause-Specific Quality of Life (MENQOL)], sleep/anxiety symptoms, serum hormone levels [e.g., follicle-stimulating hormone (FSH), luteinizing hormone (LH), and estradiol (E2)], and adverse events (AEs).

### Search Strategy and Data Extraction

Four Chinese and three English electronic databases—China biomedical literature service system (SinoMed), Wanfang database, China National Knowledge Infrastructure (CNKI), Chongqing VIP database (CQVIP), Cochrane Central Register of Controlled Trials (CENTRAL), MEDLINE (*via* PubMed), and EMBASE—with language restrictions of Chinese and English were searched from the inception date of each database until December 2020. Additional studies were also identified from other sources, including the online trial registries such as US ClinicalTrials.gov and WHO International clinical trials registry platform search portal, the reference lists of the included papers, existing systematic reviews, and gray literatures (Appendix 1).

For each study, the following data for demographic and clinical characteristics were extracted: the last name of the first author, publication year, grouping methods and number of patients in each group, duration of PMD, diagnostic criteria used, TCM syndrome type of patients, protocols including timing, frequency, and dosage in acupuncture, the acupoints selected, prescription in control group (timing, frequency, and dosage in placebo/sham acupuncture or type, dosage, and oral frequency of Western medication), outcome measures, results, follow-up, and AEs. Additionally, GetData software (Version 2.25) was used to measure the data if the outcomes were only shown graphically.

### Study Quality and Risk of Bias Assessment

Two assessors carried out independent evaluations (including determining risk of bias and assessing the internal validity) of all the included RCTs using Cochrane Collaboration's risk of bias tool (33). The revised Standards for Reporting Interventions in Clinical Trials of Acupuncture (STRICTA) checklist (revised version, published in 2010) was used to evaluate and describe the details of acupuncture procedure including completeness and reporting quality in each RCT (34).

## Data Analysis

The meta-analysis was performed *via* Cochrane Collaboration Review Manager Software (RevMan Version 5.3). The inverse-variance method in RevMan was used to assign weight to each included study. Given that the major outcome measures (e.g., global scores of depression/perimenopause scales and hormone levels) were continuous variables, mean differences (MDs) were analyzed. When the depression in different studies was assessed *via* different scales or different versions of the same scale (e.g., 17-item HAMD, 21-item HAMD, and 2-item HAMD, etc.) or when serum hormonal levels were presented in the different units of measurement, standardized MDs (SMDs) were used. Confidence intervals (CIs) were established at 95%. Level of heterogeneity across studies was tested using the *Q*- and *I*^2^-test. Statistically significance was set at two-tailed probability (*p*) value < 0.05. The results were pooled using a fixed-effects model when the *p*-value was >0.10 in the *Q*-test and the *I*^2^-value was ≤50%, which was considered be to an acceptable level of heterogeneity. Otherwise, a random-effects model was applied. When significant heterogeneity existed, subgroup analyses were carried out based on different acupuncture stimulations (MA or EA), different prescriptions in the controls (antidepressant, HRT, or antidepressant +HRT), and different versions of HAMD used. Sensitivity analysis and meta-regression analysis were also adopted to explore sources of heterogeneity and check robustness of the conclusions. Publication bias was investigated *via* Egger's test and Begg's test.

## Results Analysis

**Figure 1:**
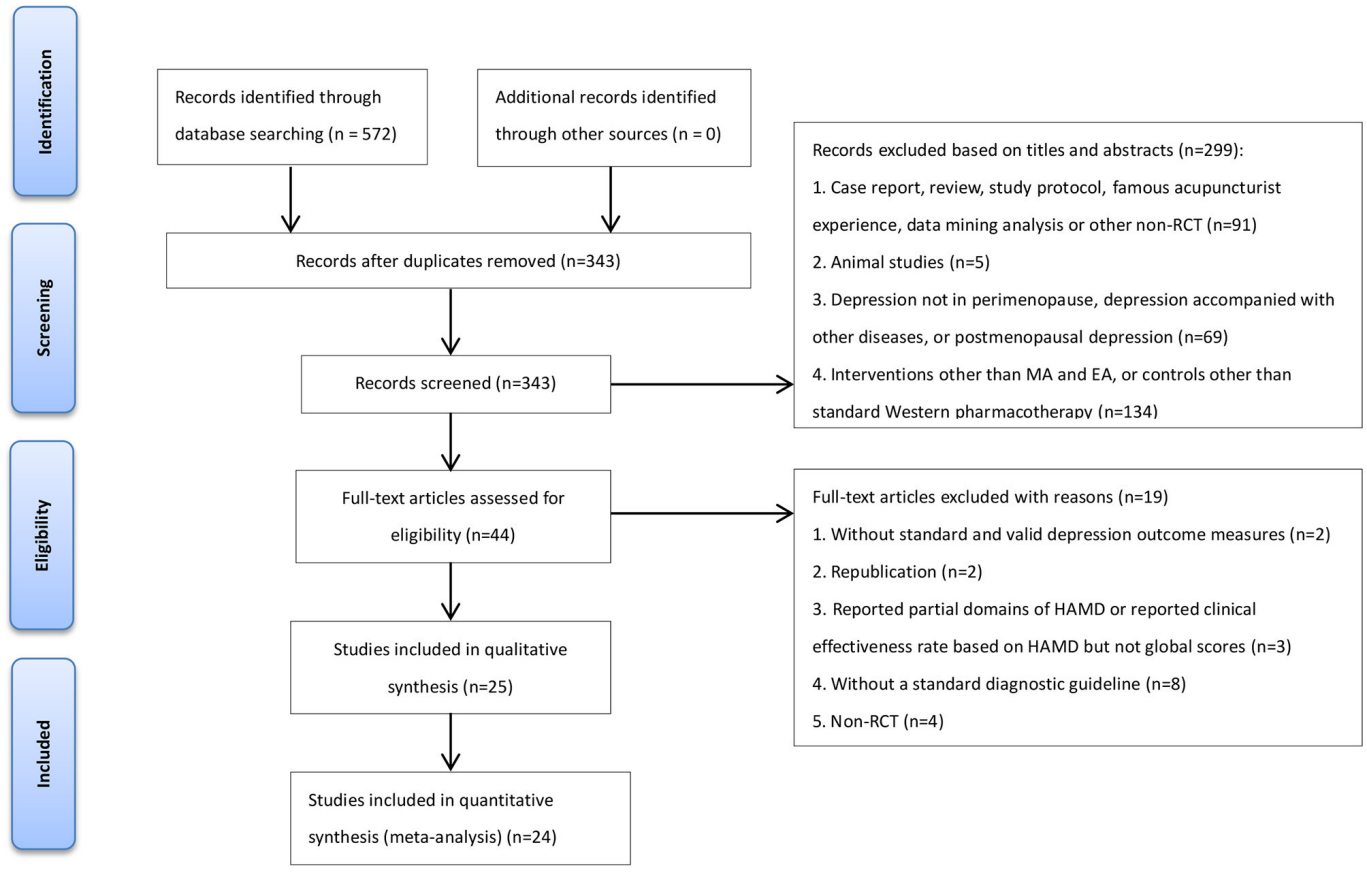
Flow diagram of the study selection process. MA, manual acupuncture; EA, electroacupuncture; RCT, randomized controlled trial.

The initial search yielded 572 potentially eligible studies. After removing the duplicates and further screening, 25 studies (involving 2,213 participants) met the predefined criteria (Figure 1). All included studies were qualitatively analyzed, and 24 of them underwent quantitative synthesis (meta-analysis).

### Description of Studies

Among the 25 RCTs, two RCTs (35, 36) employed sham-acupuncture control [one RCT (36) was a three-arm trial with both sham acupuncture and antidepressant as controls], 20 RCTs (30, 31, 37–54) employed standard care (antidepressant or antidepressant combined with HRT) control, and the remaining three (55–57) compared the clinical effectiveness between standard care (antidepressant or antidepressant combined with HRT) alone and acupuncture combined with standard care. None of the RCTs included a waitlist control. In the studies with standard care as control, the frequency of use of antidepressant drugs from high to low were fluoxetine (10/24), fluoxetine combined with HRT (5/24), escitalopram (4/24), Deanxit (3/24), sertraline (1/24), and paroxetine (1/24). None of the trials addressed the comparison between acupuncture and HRT alone. Seven out of the 25 RCTs (36, 37, 45, 46, 48, 52, 55) investigated the clinical effectiveness of EA, while the remaining 18 RCTs investigated the effectiveness of MA. Acupuncture treatment was provided daily to three times per week for 10 days−12 weeks (Table 1).

**Table 1:** Study characteristics of 25 included studies.

**References**	**Group/size**	**Age (year)**	**Depression duration (m = month, y = year)**	**Diagnostic system**	**TCM Syndrome Type**	**Acupuncture interventions**	**Acupoints**	**Prescription in control group (placebo or Western medication)**	**Outcome measure tool**	**Acupuncture/Acupuncture + Western medication compared with control (waitlist, placebo-/sham-acupuncture, Western medication)**	**Follow-up**	**Adverse events**
Wang et al. (35)	- MA/*n =* 33 - Sham-MA/*n =* 33	- MA/48.72 ± 4.21 - Sham-MA/47.82 ± 4.35	- MA/NR - Sham-MA/NR	CCMD-3	NR	30 min/day, 3 days/week for 8 weeks	KI6, LU7, PC6, SP4	sham-MA 30 min/day, 3 days/week for 8 weeks	(i) SDS (ii) MENQOL	(i) compared with sham-MA *p* < 0.05 (ii) compared with sham-MA *p* < 0.05	Lower SDS in MA in 4-week follow-up (ii) no difference in MENQOL between MA and sham-MA in 4-week follow-up	NR
Li (36)	- EA/*n =* 30 - Sham-EA/*n =* 30 - Escitalopram/*n =* 30	- EA/49.80 ± 3.39 - Sham-EA/49.83 ± 4.10 - Escitalopram/49.90 ± 2.98	- EA/30.73 ± 18.57 m - Sham-EA/30.43 ± 22.15 m - Escitalopram/26.43 ± 17.86 m	ICD-10	NR	−30 min/day, 3 days/week for 12 weeks - dense-sparse waves, 10/50 Hz, 0.5–1 mA	CV4, EX-CA1, EX-HN3, GV20, LI4, LR3, SP6, ST25	- sham-EA 30 min/day, 3 days/week for 12 weeks - Escitalopram 10 mg/day for 12 weeks	(i) HAMD (ii) MENQOL (iii) FSH (iv) E2 (v) LH	(i-i) compared with sham-EA *p* < 0.05 (i-ii) compared with Escitalopram *P* > 0.05 (ii-i) compared with sham-EA *p* < 0.05 (ii-ii) compared with Escitalopram *P* > 0.05 (iii) compared with sham-EA or Escitalopram *P* > 0.05 (iv) compared with sham-EA or Escitalopram *P* > 0.05 (v) compared with sham-EA or Escitalopram *P* > 0.05	(i) Lower HAMD in EA in 4- and 12- week follow-up (ii) Lower MENQOL in acupuncture in 4-, 8-, and 12- week follow-up	- EA/*n =* 2 [hematoma] - sham-EA/*n =* 0 - - Escitalopram/*n =* 25 [fatigue (17); headache (2); sleep disturbance (7); dizziness (7); palpitation (4); sweating (10); dry mouth (14); constipation (8)]
Li et al. (37)	- EA/*n =* 116 - Escitalopram/*n =* 105	- EA/49.83 ± 3.10 - Escitalopram/49.93 ± 3.10	- EA/20.60 ± 16.20 m - Escitalopram/20.20 ± 16.50 m	DSM-V, ICD-10	NR	−30 min/day, 3 days/week for 12 weeks - dilatational wave wave, 50 Hz, 0.5–1 mA	CV4, EX-CA1, EX-HN3, GV20, LI4, LR3, SP6, ST25	- Escitalopram 10 mg/day for 12 weeks	(i) HAMD (ii) MENQOL (iii) FSH (iv) E2 (v) LH	(i) compared with Escitalopram *P* > 0.05 (ii) compared with Escitalopram *P* > 0.05 (iii) compared with Escitalopram *P* > 0.05 (iv) compared with Escitalopram *P* > 0.05 (v) compared with Escitalopram *P* > 0.05	(i) Lower HAMD in EA in 4- and 12- week follow-up (ii) Lower MENQOL in acupuncture in 4-, 8- and 12- week follow-up	- EA/*n =* 14 [hematoma] - Escitalopram/*n =* 18 [dizziness, palpitation, stomachache]
Chi and Zou (30)	- MA/*n =* 30 - Fluoxetine/*n =* 30	- MA/51.63 ± 1.72 - Fluoxetine/51.43 ± 1.62	- MA/10.61 ± 6.10 m - Fluoxetine/11.12 ± 5.58 m	CCMD-3	NR	30 min/day for 4 weeks	EX-HN1, EX-HN3, GV20, KI3, LR3, LR14, SP6, ST36	- Fluoxetine 20 mg/day for 4 weeks	(i) HAMD	(i) compared with Fluoxetine *p* < 0.05	No follow-up	- MA/*n =* 0 - Fluoxetine/*n =* 3 [dizziness (1); nausea (2)]
Deng (38)	- MA/*n =* 29 - Deanxit/*n =* 29	- MA/50.03 ± 4.43 - Deanxit/48.70 ± 4.93	- MA/6.25 ± 2.31 m - Deanxit/5.77 ± 3.64 m	ICD-10	NR	20–30 min/day, after 3 consecutive days of treatment, once treatment every 3 days for total 4 weeks	CV3, CV4, CV6, CV10, CV12, KI17, Qipang (0.5 *Cun* beside CV6), Xiafengshidian (1 *Cun* below and beside ST26)	- Deanxit 20 mg/day for 4 weeks	(i) HAMD (ii) KI (iii) 5-HT	(i) compared with Deanxit *P* > 0.05 (ii) compared with Deanxit *P* > 0.05 (iii) compared with Deanxit *P* > 0.05	(i) Lower HAMD in MA in 2- week follow-up (ii) no difference in HAMD between MA and Deanxit in 4- week follow-up (iii) no difference in KI between MA and Deanxit in 2- and 4- week follow-up	- MA/*n =* 3 [changes of character of stool (2); palpitation (1)]- Deanxit/*n =* 32 [changes of character of stool (6); dry mouth and halitosis (9); dysphoria (6); dreaminess (6); breast distending pain (5)]
Dong (39)	- MA/*n =* 30 - Nilestriol + Fluoxetine/*n =* 30	- MA/55.00 - Nilestriol + Fluoxetine/53.00	- MA/24.00 m - Nilestriol + Fluoxetine/26.00 m	CCMD-3, CDTE-TCM	NR	30 min/day for 30 days	BL13, BL15, BL17, BL18, BL20, BL21, BL23	Nilestriol 2 mg/15 days for 30 days + Fluoxetine 20 mg/day for 30 days	(i) HAMD	(i) compared with Nilestriol + Fluoxetine *p* < 0.05	No follow-up	NR
Li (40)	- MA/*n =* 32 - Fluoxetine/*n =* 32	- MA/50.59 ± 2.94 - Fluoxetine/50.25 ± 2.71	- MA/20.63 ± 7.49 m - Fluoxetine/20.31 ± 7.45 m	CCMD-3	*Liver* stagnation and *Kidney* deficiency	30 min/day, 6 days/week for 12 weeks	BL15, BL18, BL23, EX-HN1, EX-HN3, GV20, GV24, PC6	20 mg/day for 12 weeks	(i) HAMD (ii) KI	(i) compared with Fluoxetine *p* < 0.05 (ii) compared with Fluoxetine *p* < 0.05	follow-up for 12 weeks; no data of HAMD and KI total scores for follow-up	- MA/*n =* 0- Fluoxetine/ *n =* 8 [nausea or vomiting (2); dry mouth (1); indigestion (1); diarrhea (1); dizziness (1); headache (1)]
Ma and Liu (41)	- MA/*n =* 30 - Fluoxetine/*n =* 30	- MA/53.45 ± 4.82 - Fluoxetine/52.74 ± 5.17	- MA/8.24 ± 4.76 m - Fluoxetine/7.65 ± 4.52 m	CCMD-3, CDTE-TCM	NR	30 min/day, 5 days/week for 8 weeks	EX-HN1, EX-HN3, GV20, HT7, PC6, PC7, SP6, ST36	20 mg/day for 8 weeks	(i) HAMD	(i) compared with Fluoxetine *P* > 0.05	No follow-up	- MA/*n =* 0- Fluoxetine/ *n =* 6 [nausea (2); dizziness (2)]
Niu and Wang (42)	- MA/*n =* 41 - Fluoxetine/*n =* 41	- MA/54.10 ± 2.00 - Fluoxetine/54.20 ± 2.10	- MA/7.70 ± 1.20 m - Fluoxetine/7.70 ± 1.30 m	CCMD-3	Stagnation of *Liver*-Qi	30 min/day, 5 days/week for 6 weeks	BL13, BL15, BL17, BL18, BL20, BL23	20 mg/day for 6 weeks	(i) HAMD	(i) compared with Fluoxetine *p* < 0.05	No follow-up	- MA/*n =* 7 [dizziness (2); palpitation (1); dry mouth (1); nausea (3)] - Fluoxetine/*n =* 6 [dizziness (1); palpitation (2); dry mouth (2); nausea (1)]
Qian et al. (43)	- MA/*n =* 33 - Fluoxetine/*n =* 30	- MA/54.00 - Fluoxetine/55.00	- MA/7.00 m - Prozac/8.00 m	CCMD-3	NR	25 min/day, 5 days/week for 6 weeks	BL13, BL15, BL17, BL18, BL20, BL23	20 mg/day for 6 weeks	(i) HAMD	(i) compared with Fluoxetine *P* > 0.05	No follow-up	- MA/*n =* 2 [dizziness (1); palpitation (1)] - Fluoxetine/*n =* 9 [insomnia (1); akathisia (1); dry mouth (1); nausea (1); palpitation (1); skin symptom (1); forexcitation and agitation (2)]
Qiang (44)	- MA/*n =* 30 - Fluoxetine/*n =* 30	- MA/54.32 ± 3.29 - Fluoxetine/54.00 ± 4.62	- MA/11.32 ± 6.25 m - Fluoxetine/12.12 ± 4.58 m	CCMD-3	NR	25 min/day, 5 days/week for 6 weeks	BL15, BL18, BL23,EX-HN1, GB20	20 mg/day for 6 weeks	(i) HAMD	(i) compared with Prozac *P* > 0.05	No follow-up	NR
Shi et al. (45)	- EA/*n =* 30 - Escitalopram/*n =* 30	- EA/48.70 ± 1.99 - Escitalopram/49.43 ± 1.87	- EA/14.17 ± 4.99 m - Escitalopram/14.23 ± 5.58 m	DSM-V	NR	−30 min/day, 3 days/week for 12 weeks- dense-sparse waves, 10/50 Hz, 0.5–1.0 mA	CV4, EX-CA1, EX-HN3, GV20, LI4, LR3, SP6, ST25	10 mg/day for 12 weeks	(i) HAMD	(i) compared with Escitalopram *p* < 0.05	(i) Lower HAMD in EA in 4- and 12- week follow-up	NR
Sun et al. (46)	- EA/*n =* 21 - Escitalopram/*n =* 21	- EA/50.29 ± 2.59 - Escitalopram/49.86 ± 3.83	- EA/1.94 ± 0.68 m - Citalopram/1.56 ± 0.94 m	DSM-V	NR	−30 min/day, 3 days/week for 12 weeks - dense-sparse waves, 10/50 Hz, 0.5–1.0 mA	CV4, EX-CA1, EX-HN3, GV20, LI4, LR3, SP6, ST25	10 mg/day for 12 weeks	(i) HAMD	(i) compared with Escitalopram *p* < 0.05	No follow-up	NR
Wang et al. (47)	- MA/*n =* 21 - Deanxit/*n =* 21	- MA/49.60 ± 4.30 - Deanxit/48.30 ± 4.70	NR	CCMD-3	NR	−30 min/day, after 3 consecutive days of treatment, once treatment every 3 days for total 4 weeks	CV3, CV4, CV6, CV10, CV12, KI17	10 mg/day for 4 weeks	(i) HAMD	(i) compared with Prozac *P* > 0.05	(i) Lower HAMD in MA in 2- and 4- week follow-up	- MA/*n =* 3 [changes of character of stool (2); palpitation (1)] - Deanxit/*n =* 15 [dry mouth and halitosis (9); dysphoria, dreaminess or breast distending pain (6)]
Zhang (48)	- EA/*n =* 44 - Nilestriol+ Fluoxetine/*n =* 46	- EA/48.48 ± 5.39 - Nilestriol+ Fluoxetine/48.16 ± 4.15	- EA/29.15 ± 25.90 m - Nilestriol+ Fluoxetine/27.45 ± 28.83 m	CCMD-3	NR	−30 min/day, 5 days/week for 12 weeks - dilatational wave wave, 8–9 mA, 6 V	BL13, BL15, BL17, BL20, BL23, GV20, KI3, LR3, PC6, SP6	Nilestriol 2 mg/14 days for 30 days + Fluoxetine 20 mg/day for 12 weeks	(i) HAMD (ii) KI (iii) FSH (iv) E2 (v) LH	(i) compared with Nilestriol+ Fluoxetine *P* > 0.05 (ii) compared with Nilestriol+ Fluoxetine *P* > 0.05 (iii) compared with Nilestriol+ Fluoxetine *P* > 0.05 (iv) compared with Nilestriol+ Fluoxetine *P* > 0.05 (v) compared with Nilestriol+ Fluoxetine *P* > 0.05	No follow-up	- EA/*n =* 5 [sweating, dizziness, vomiting] - Nilestriol+ Fluoxetine/*n =* 23 [dry mouth and halitosis (5); nausea (6); dysphoria (2); constipation (6); dreaminess (2); breast distending pain (2)]
Zhang (49)	- MA/*n =* 94 - Premarin + Provera + Fluoxetine/*n =* 94	- MA/50.10 ± 2.70 - Premarin + Provera + Fluoxetine/49.80 ± 2.60	- MA/1.40 ± 0.50y - Premarin + Provera + Fluoxetine/1.30 ± 0.40y	CCMD-3	NR	30 min/day, 7 days/week for 12 weeks	EX-HN1, GB13, GV20, GV24, HT7	Premarin 0.625 mg/day and Provera 6 mg/day + Fluoxetine 20 mg/day for 12 weeks	(i) HAMD (ii) FSH (iii) E2 (iv) LH	(i) compared with Premarin + Provera + Fluoxetine *p* < 0.05 (ii) compared with Premarin + Provera + Fluoxetine *P* > 0.05 (iii) compared with Premarin + Provera + Fluoxetine *P* > 0.05 (iv) compared with Premarin + Provera + Fluoxetine *P* > 0.05	No follow-up	- MA/*n =* 2 [feeling pain when inserting needle] - Premarin + Provera + Fluoxetine/*n =* 12 [dizziness (5); nausea and vomiting (4); hypersomnia (3)]
Zheng et al. (50)	- MA/*n =* 60 - Premarin + Provera + Fluoxetine/*n =* 60	- MA/52.27 ± 3.45 - Premarin + Provera + Fluoxetine/51.98 ± 3.14	- MA/1.22 ± 0.87y - Premarin + Provera + Fluoxetine/1.34 ± 0.92y	CCMD-3	NR	30 min/day, 7 days/week for 12 weeks (needle retaining time for 8 h in BL8, GV19, GV21 per session)	BL8, BL18, BL23, GV19, GV21, KI3, LR3, SP6	Premarin 0.625 mg/day for 20 days + and Provera 6 mg/day + Fluoxetine 20 mg/day for 12 weeks	(i) HAMD (ii) KI (iii) FSH (iv) E2 (v) LH	(i) compared with Premarin + Provera + Fluoxetine *P* > 0.05(ii) compared with Premarin + Provera + Fluoxetine *P* > 0.05 (iii) compared with Premarin + Provera + Fluoxetine *P* > 0.05 (iv) compared with Premarin + Provera + Fluoxetine *p* < 0.05 (v) compared with Premarin + Provera + Fluoxetine *P* > 0.05	(i) Lower HAMD and KI in MA in 24- week follow-up	- MA/*n =* 2 [feeling pain when inserting needle] - Premarin + Provera + Fluoxetine/*n =* 18 [loss of appetite (5); dizziness (4); diarrhea (3); breast distending pain (3); leukorrhagia (2); spasmus (1)]
Ding and Liu (51)	- MA/*n =* 39 - Fluoxetine/*n =* 39	- MA/49.68 ± 3.90 - Fluoxetine/49.50 ± 3.51	NR	CCMD-2-R	NR	30 min/day, 6 days/week for 4 weeks	BL15, BL18, BL20, BL23, GV20, HT7, LR3, SP6	20 mg/day for 4 weeks	(i) HAMD (ii) KI	(i) compared with Fluoxetine *P* > 0.05 (ii) compared with Fluoxetine *p* < 0.05	No follow-up	NR
Li and Dai (52)	- EA/*n =* 30 - Fluoxetine/*n =* 30	NR	NR	CDTE-TCM	NR	−25 min/day, 3 days/week for 6 weeks - dilatational wave, 15 Hz, 1 mA	EX-HN1, EX-HN3, GV20, HT7, LI4, PC6, SP6, ST36	20 mg/day for 6 weeks	(i) HAMD (ii) HAMA	(i) compared with Fluoxetine *P* > 0.05 (ii) compared with Fluoxetine *p* < 0.05	No follow-up	NR
Zhang (53)	- MA/*n =* 29 - Deanxit/*n =* 29	NR	NR	CCMD-3	NR	30 min/day, after 3 consecutive days of treatment, once treatment every 3 days for total 4 weeks	CV3, CV4, CV6, CV10, CV12, KI17	20 mg/day for 4 weeks	(i) HAMD	(i) compared with Deanxit *P* > 0.05	(i) Lower HAMD in MA in 2- and 4- week follow-up	NR
Xing (54)	- MA/*n =* 120 - Fluoxetine/*n =* 120	- MA/51.20 ± 5.40 - Fluoxetine/49.50 ± 6.80	- MA/11.60 ± 7.30 m - Fluoxetine/10.50 ± 8.60 m	CCMD-3	Stagnation of *Liver*-Qi, *Heart* and *Spleen* deficiency; *Liver* depression and phlegm-heat	20 min/day, 7 days/week for 6 weeks	GV26, PC5	20 mg/day for 6 weeks	(i) HAMD	(i) compared with Fluoxetine *P* > 0.05	No follow-up	NR
Zhou and Wu (31)	- MA/*n =* 30 - Fluoxetine/*n =* 28	- MA/51.80 ± 4.20 - Fluoxetine/48.90 ± 3.80	- MA/30.63 ± 10.12 m - Prozac/26.33 ± 9.65 m	CCMD-3	*Liver* and *kidney Yin* deficiency, *Spleen* and *Kidney* Yang deficiency, stagnated Qi transforming into fire, stagnation of phlegm and Qi	30 min/day, 6 days/week for 6 weeks	BL15, BL18, BL23, EX-HN1, GB13, GV24, SP6, ST36	20 mg/day for 6 weeks	(i) HAMD (ii) 5-HIAA (iii) NE (iv) DA	(i) compared with Fluoxetine *p* < 0.05 (ii) compared with Fluoxetine *P* > 0.05 (iii) compared with Fluoxetine *P* > 0.05 (iv) compared with Fluoxetine *p* < 0.05	No follow-up	NR
Ma et al. (55)	- EA + Paroxetine/*n =* 55 - Paroxetine/*n =* 50	- EA + Paroxetine /52.95 ± 5.86 - Paroxetine /51.49 ± 6.03	- EA + Paroxetine/5.49 ± 4.86 m - Paroxetine/4.98 ± 4.75 m	CCMD-3	NR	−45 min/day, 7 days/week for 6 weeks - dilatational wave wave, 8–9 mA	EX-HN3, GV20, LI4, PC6, ST36	−10 mg/day for 6 weeks	(i) HAMD	(i) compared with Paroxetine *P* > 0.05	No follow-up	- EA + Paroxetine/*n =* 2 [dizziness (1); nausea (1)] - Paroxetine/*n =* 3 [dizziness (1); elevated blood pressure (2)]
Liu and Chen (56)	- MA + Sertraline/*n =* 40 - Sertraline/*n =* 40	- MA + Sertraline/51.50 ± 3.40 - Sertraline/52.10 ± 3.30	- MA + Sertraline/2.58 ± 2.18y - Sertraline/2.67 ± 1.73y	- CCMD-3, - ICD-10	NR	30 min/day, 3 days/week for 12 weeks	BL23, CV4, HT7, KI3, LI4, LR3, SP6	50 mg/day for 6 weeks	(i) HAMD (ii) FSH (iii) E2 (iv) 5-HT (v) GABA	(i) compared with Sertraline *p* < 0.05 (ii) compared with Sertraline *p* < 0.05 (iii) compared with Sertraline *p* < 0.05 (iv) compared with Sertraline *p* < 0.05 (v) compared with Sertraline *p* < 0.05	No follow-up	NR
Ning (57)	- MA + Nilestriol + Fluoxetine/*n =* 45 - Nilestriol + Fluoxetine/*n =* 45	NR	NR	Psychiatry textbook	NR	30 min/day, 7 days/week for 12 weeks	BL13, BL15, BL18, BL20, BL23, GV20, HT7, KI3, LI4, LR3	Nilestriol 2 mg/15 days + Fluoxetine 20 mg/day for 12 weeks	(i) HAMD (ii) TESS	(i) compared with Nilestriol + Fluoxetine *p* < 0.05 (ii) compared with Nilestriol + Fluoxetine *p* < 0.05	No follow-up	NR

Table 2 and Appendix 2 show the assessment time-points and results of each outcome in each trial. Except for one study (35) that adopted SDS as the primary outcome, the remaining studies adopted HAMD to assess the changes in depression at pre- and post-treatment. HAMD-17 was used in 11 RCTs (36–38, 40, 43–45, 47, 50, 52, 53), HAMD-24 in seven (30, 39, 48, 49, 51, 54, 55), and HAMD-21 in one (31). The remaining five studies (41, 42, 46, 56, 57) did not report which version of HAMD was used. KI (38, 40, 48, 50, 51) and MENQOL (35–37) were used to evaluate patients' perimenopausal symptoms and quality of life during perimenopause, respectively. In addition, serum FSH (36, 37, 48–50, 56), E2 (36, 37, 48–50, 56), and LH (36, 37, 48–50) were assessed in some RCTs to explore the association between the effect of acupuncture and the modulation of reproductive hormone levels.

**Table 2:** Trends of major outcomes for depression and perimenopausal symptoms in acupuncture (OR acupuncture + antidepressant/HRT) and comparison with controls in each study.

**References**	**Comparison**		**Outcome measures for depression**	**Outcome measures for perimenopausal symptoms**	**Sex hormone levels**		
			**HAMD/SDS**	**KI/MENQOL**	**FSH**	**E2**	**LH**
Wang et al. (35)	Vs. same group at different time-points	Post- vs. pre-treatment	↓	↓	/	/	/
		4-week follow-up vs. pre-treatment	↓	↓	/	/	/
	Acup vs. sham Acup at same time-point	Post-treatment	<	<	/	/	/
		4-week follow-up	<	(–)	/	/	/
Li (36)	Vs. same group at different time-points	Post- vs. pre-treatment	↓	↓	(–)	↓	(–)
		4-week follow-up vs. pre-treatment	↓	↓	/	/	/
		8-week follow-up vs. pre-treatment	/	↓	/	/	/
		12-week follow-up vs. pre-treatment	↓	↓	/	/	/
	Acup vs. sham Acup at same time-point	Post-treatment	<	<	(–)	(–)	(–)
		4-week follow-up	<	<	/	/	/
		8-week follow-up	/	<	/	/	/
		12-week follow-up	<	<	/	/	/
	Acup vs. antidepressant at same time-point	Post-treatment	(–)	(–)	(–)	(–)	(–)
		4-week follow-up	(–)	<	/	/	/
		8-week follow-up	/	<	/	/	/
		12-week follow-up	(–)	<	/	/	/
Li et al. (37)	Vs. same group at different time-points	Post- vs. pre-treatment	↓	↓	(–)	(–)	(–)
	Acup vs. antidepressant at same time-point	Post-treatment	(–)	(–)	(–)	(–)	(–)
		4-week follow-up	<	<	/	/	/
		8-week follow-up	/	<	/	/	/
		12-week follow-up	<	<	/	/	/
Chi and Zou (30)	Vs. same group at different time-points	Post- vs. pre-treatment	↓	/	/	/	/
	Acup vs. antidepressant at same time-point	Post-treatment	<	/	/	/	/
Deng (38)	Vs. same group at different time-points	Post- vs. pre-treatment	↓	↓	/	/	/
	Acup vs. antidepressant at same time-point	Post-treatment	(–)	(–)	/	/	/
		2-week follow-up	<	(–)	/	/	/
		4-week follow-up	(–)	(–)	/	/	/
Dong (39)	Vs. same group at different time-points	Post- vs. pre-treatment	↓	/	/	/	/
	Acup vs. HRT + antidepressant at same time-point	Post-treatment	<	/	/	/	/
Li (40)	Vs. same group at different time-points	Post- vs. pre-treatment	↓	↓	/	/	/
	Acup vs. antidepressant at same time-point	Post-treatment	<	<	/	/	/
Ma and Liu (41)	Vs. same group at different time-points	Post- vs. pre-treatment	↓	/	/	/	/
	Acup vs. antidepressant at same time-point	Post-treatment	(–)	/	/	/	/
Niu and Wang (42)	Vs. same group at different time-points	Post- vs. pre-treatment	↓	/	/	/	/
	Acup vs. antidepressant at same time-point	Post-treatment	<	/	/	/	/
Qian et al. (43)	Vs. same group at different time-points	Post- vs. pre-treatment	↓	/	/	/	/
	Acup vs. antidepressant at same time-point	Post-treatment	(–)	/	/	/	/
Qiang (44)	Vs. same group at different time-points	Post- vs. pre-treatment	↓	/	/	/	/
	Acup vs. antidepressant at same time-point	Post-treatment	(–)	/	/	/	/
Shi et al. (45)	Vs. same group at different time-points	Post- vs. pre-treatment	↓	/	/	/	/
	Acup vs. antidepressant at same time-point	Post-treatment	<	/	/	/	/
		4-week follow-up	<	/	/	/	/
		12-week follow-up	<	/	/	/	/
Sun et al. (46)	Vs. same group at different time-points	Post- vs. pre-treatment	↓	/	/	/	/
	Acup vs. antidepressant at same time-point	Post-treatment	<	/	/	/	/
Wang et al. (47)	Vs. same group at different time-points	Post- vs. pre-treatment	↓	/	/	/	/
	Acup vs. antidepressant at same time-point	Post-treatment	(–)	/	/	/	/
		2-week follow-up	<	/	/	/	/
		4-week follow-up	<	/	/	/	/
Zhang (48)	Vs. same group at different time-points	Post- vs. pre-treatment	↓	↓	↓	↑	↓
	Acup vs. HRT + antidepressant at same time-point	Post-treatment	(–)	(–)	(–)	(–)	(–)
Zhang (49)	Vs. same group at different time-points	Post- vs. pre-treatment	↓	/	↓	↑	↓
	Acup vs. HRT + antidepressant at same time-point	Post-treatment	<	/	(–)	(–)	(–)
Zheng et al. (50)	Vs. same group at different time-points	Post- vs. pre-treatment	↓	↓	↓	↑	↓
	Acup vs. HRT + antidepressant at same time-point	Post-treatment	(–)	(–)	(–)	<	(–)
		24-week follow-up	<	<	/	/	/
Ding and Liu (51)	Vs. same group at different time-points	Post- vs. pre-treatment	↓	↓	/	/	/
	Acup vs. antidepressant at same time-point	Post-treatment	(–)	<	/	/	/
Li and Dai (52)	Vs. same group at different time-points	Post- vs. pre-treatment	↓	/	/	/	/
	Acup vs. antidepressant at same time-point	Post-treatment	(–)	/	/	/	/
Zhang (53)	Vs. same group at different time-points	Post- vs. pre-treatment	↓	/	/	/	/
	Acup vs. antidepressant at same time-point	Post-treatment	(–)	/	/	/	/
		2-week follow-up	<	/	/	/	/
		4-week follow-up	<	/	/	/	/
Xing (54)	Vs. same group at different time-points	Post- vs. pre-treatment	↓	/	/	/	/
	Acup vs. antidepressant at same time-point	Post-treatment	(–)	/	/	/	/
Zhou and Wu (31)	Vs. same group at different time-points	Post- vs. pre-treatment	↓	/	/	/	/
	Acup vs. antidepressant at same time-point	post-treatment	(–)	/	/	/	/
Ma et al. (55)	Vs. same group at different time-points	Post- vs. pre-treatment	↓	/	/	/	/
	Acup + antidepressant vs. antidepressant at same time-point	Post-treatment	(–)	/	/	/	/
Liu and Chen (56)	Vs. same group at different time-points	Post- vs. pre-treatment	↓	/	↓	↑	/
	Acup + antidepressant vs. antidepressant at same time-point	Post-treatment	<	/	<	<	/
Ning (57)	Vs. same group at different time-points	Post- vs. pre-treatment	↓	/	/	/	/
	Acup + antidepressant vs. antidepressant at same time-point	Post-treatment	<	/	/	/	/

*↑, statistically increase; ↓, statistically decrease; >, statistically higher/longer/more; <, statistically lower/shorter/less; (–), no statistical difference/no statistical changes; Acup, acupuncture; HRT, hormone replacement therapy; HAMD, Hamilton Depression Scale; SDS, Self-Rating Depression Scale; KI, Kupperman index; MENQOL, Menopause-Specific Quality of Life; FSH, follicle-stimulating hormone; LH, luteinizing hormone; E2, estradiol*.

Eight studies (35–38, 45, 47, 50, 53) reported follow-up data from 2 to 24 weeks after the end of treatment (Appendix 2).

Thirteen studies (30, 36–38, 40–43, 47–50, 55) reported AEs. AEs associated with acupuncture treatment were hematoma (16/146), feeling pain when inserting needle (4/154), dizziness (8/147), palpitation (4/83), dry mouth (1/41), nausea and/or vomiting (6/85), changes of character of stool (4/50), and sweating (5/44); AEs associated with standard (antidepressant or antidepressant combined with HRT) included fatigue (17/30), palpitation (25/206), headache (3/62), dizziness (40/472), sleep disturbance (25/250), sweating (10/30), dry mouth and/or halitosis (41/229), indigestion (1/32), loss of appetite (5/60), stomachache (18/105), nausea and/or vomiting (18/303), constipation (14/76), diarrhea (4/92), changes of character of stool (6/29), dysphoria (14/96), for excitation and agitation (2/30), akathisia (1/30), spasmus (1/60), breast distending pain (16/156), leukorrhagia (2/60), skin symptom (1/30), and elevated blood pressure (2/50). No sham-acupuncture-related AEs were reported (Table 1).

### Study Quality Evaluation

**Figure 2:**
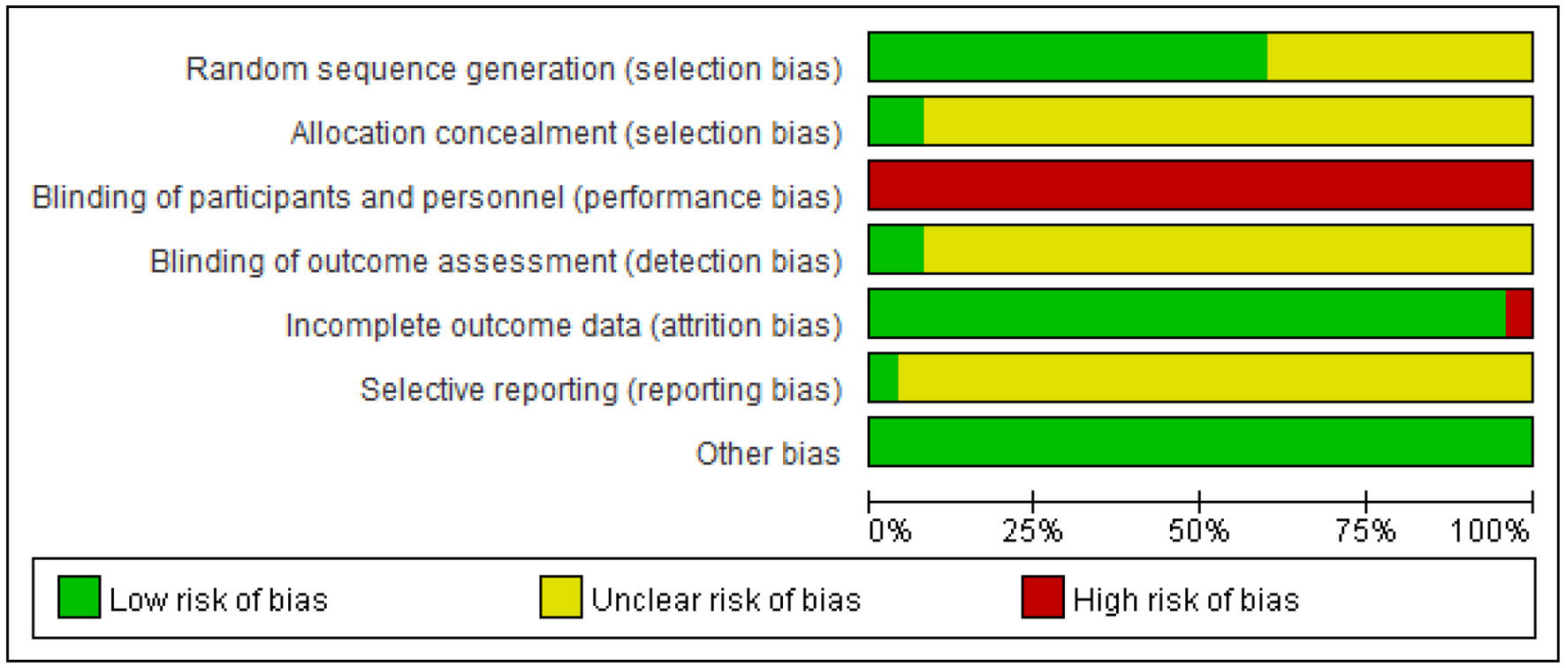
Risk of bias summary. Other biases are assessed based on baseline balance.

Fifteen out of 25 trials provided an adequate description of the process and method of randomization (31, 35–38, 42–44, 47, 48, 50, 52, 54, 56, 57), while 10 trials (30, 39–41, 45, 46, 49, 51, 53, 55) only mentioned that the RCT design was employed in the trial but did not clarify the specific randomization procedure. All except for two trials were judged as being unclear in risk of bias in the domain of allocation concealment (30, 31, 35, 38–57). Only two trials (36, 37) reported blinding of outcome assessment. Incomplete outcome data were judged as low risk of bias in 24 studies. Amongst them, 16 studies (30, 37, 39, 41, 42, 44–46, 49–54, 56, 57) reported no withdrawal of patients. In the remaining eight studies (31, 35, 36, 38, 40, 43, 47, 55), the dropout cases in each study were <10% of the initial samples, which is within the controllable range. For the item of selective outcome reporting, one RCT (37) was assessed as low risk of bias, as its protocol was registered in the ChiCTR. The remaining studies were rated as unclear risk of bias because of unavailable protocols or there was insufficient evidence and information to permit a clear judgment. “Blinding of personnel (acupuncturist)” in all studies was rated as a high risk of bias due to the nature of acupuncture. Acupuncture techniques require manipulation by a qualified professional to perform; thus, it is not feasible to blind the trial acupuncturists. In two studies with sham acupuncture as control, participants (patients) were blinded and clearly described in one study (36). All RCTs addressed baseline balance adequately (Figure 2, Appendices 3, 4).

Appendix 5 summarizes the details about acupuncture per STRICTA guideline. Traditional Chinese acupuncture was used in all 25 studies, and treatment was provided in accordance with TCM theory. As the core part of acupuncture therapy, the needling details were not clearly reported in all RCTs. For instance, the depth of insertion was presented in detail in only 16 trials (30, 31, 36, 40–46, 49–52, 54, 57), and four studies did not show the needle type used (35, 49, 53, 55). All trials gave the information of the needle retention time ranging from 20 to 45 min. Setting of treatment was not illustrated in any included trial. Only one RCT (37) introduced acupuncturist's background.

### Analysis of Outcome Measures

The qualitative and quantitative analyses for outcome measures in the 25 included studies were divided into three parts: (1) acupuncture vs. sham acupuncture (*n* = 2); (2) acupuncture vs. Western medicine (antidepressant or antidepressant combined with HRT) (*n* = 21); and (3) acupuncture combined with Western medicine vs. Western medicine (*n* = 3). One RCT had three arms with acupuncture vs. sham acupuncture vs. antidepressant (Appendix 6).

#### Acupuncture vs. Sham Acupuncture

Two studies (35, 36) (*n* = 126) were under this category and used HAMD and SDS as the primary outcome, respectively. Both studies found that acupuncture significantly reduced the global scores of HAMD/SDS and MENQOL, in comparison with sham acupuncture. These findings suggest that acupuncture can improve both the depressed mood and quality of life in women with PMD.

During the follow-up, one study (36) found that the HAMD score continued to decline, and another study (35) found SDS score slightly increased but was still significantly lower than baseline data in the acupuncture group. Both studies found HAMD/SDS of the sham-acupuncture group almost returned to the baseline level during the follow-up. One of the studies (36) investigated the impacts of acupuncture on sex hormone levels but did not find any statistically significant difference between pre- and post-treatment (Table 2).

#### Acupuncture vs. Antidepressant/Antidepressant + HRT

Twenty-one trials (*n* = 1,842) were included in this comparison. Meta-analyses were performed for five indicators, namely, HAMD, KI, FSH, E2, and LH. We did not carry out the meta-analysis for other outcome measures because there were fewer than three studies for each of them (Appendix 2).

##### Depression Symptoms

**(1) Post-Treatment**

**Figure 3:**
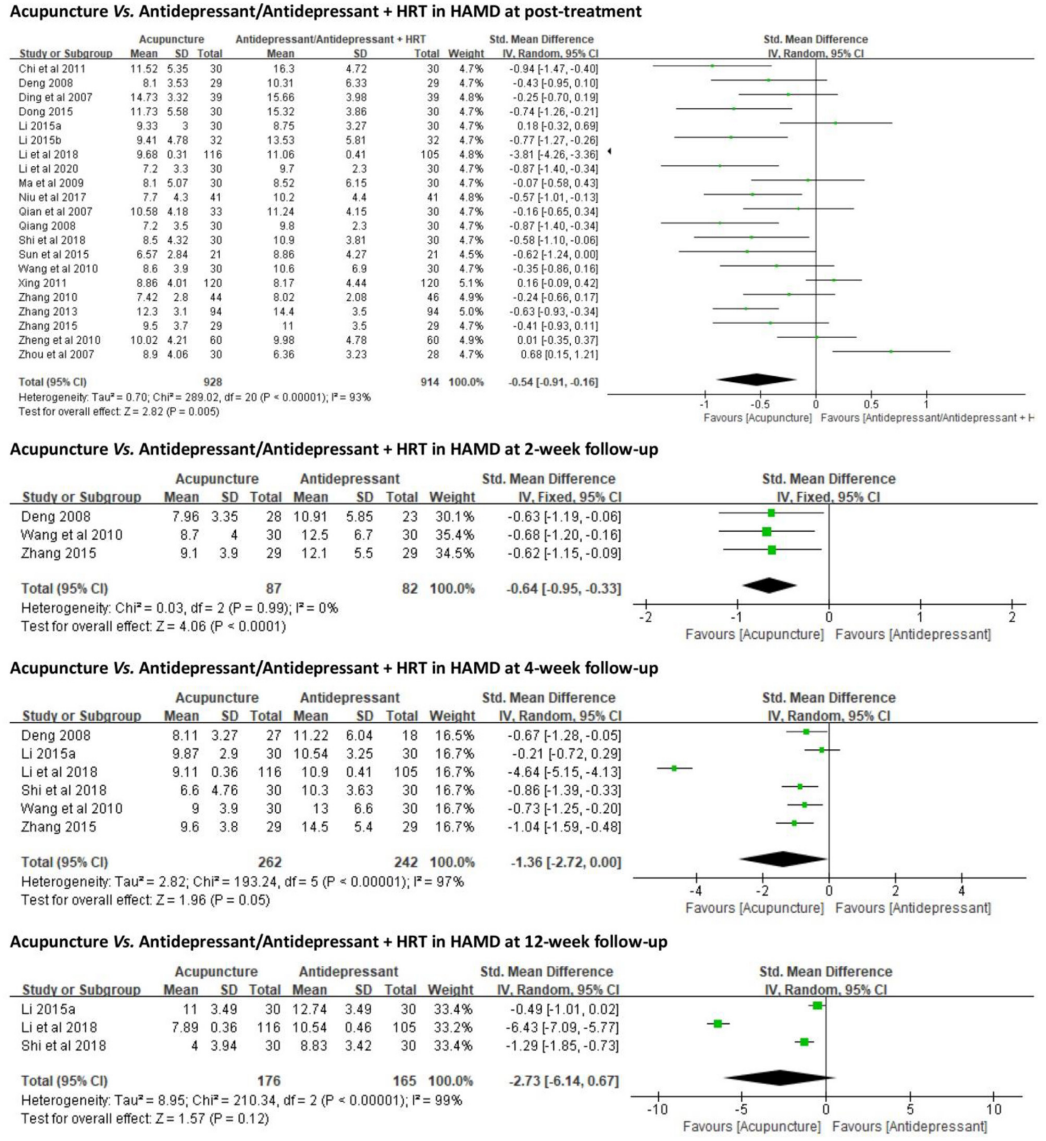
Forest plots of acupuncture vs. antidepressant/antidepressant + HRT in HAMD. HRT, hormone replacement therapy; HAMD, Hamilton Depression Scale.

All 21 trials employed HAMD as an outcome measure. Due to the high heterogeneity (*p* < 0.01, *I*^2^ = 93%), a random-effects model was used. The results favored acupuncture in reducing HAMD global scores [SMD = −0.54, 95% CI (−0.91, −0.16), *p* < 0.01] (Figure 3).

**(2) Follow-Up**

*2-Week Follow-Up*. Three (38, 47, 53) out of 21 trials compared antidepressants (Deanxit). Due to no evident heterogeneity (*p* = 0.99, *I*^2^ = 0), a fixed-effects model was used. At 2-week follow-up, the results favored acupuncture in reducing HAMD global scores [SMD = −0.64, 95% CI (−0.95, −0.33), *p* < 0.01] (Figure 3).

*4-Week Follow-Up*. Six (36–38, 45, 47, 53) out of 21 trials compared antidepressants (escitalopram or Deanxit). Due to the high heterogeneity (*p* < 0.01, *I*^2^ = 97%), a random-effects model was used. At 4-week follow-up, no significant difference was identified between acupuncture and antidepressant in reducing HAMD global scores [SMD = −1.36, 95% CI (−2.72, 0.00), *p* = 0.05] (Figure 3).

*12-Week Follow-Up. Three* (36, 37, 45) out of 21 trials compared antidepressants (escitalopram). A random-effects model was used due to the high heterogeneity (*p* < 0.01, *I*^2^ = 99%). At 12-week follow-up, there was no significant difference between acupuncture and antidepressant in reducing HAMD global scores [SMD = −2.73, 95% CI (−6.14, 0.67), *p* = 0.12] (Figure 3).

##### Perimenopausal Symptoms and Hormonal Levels

**Figure 4:**
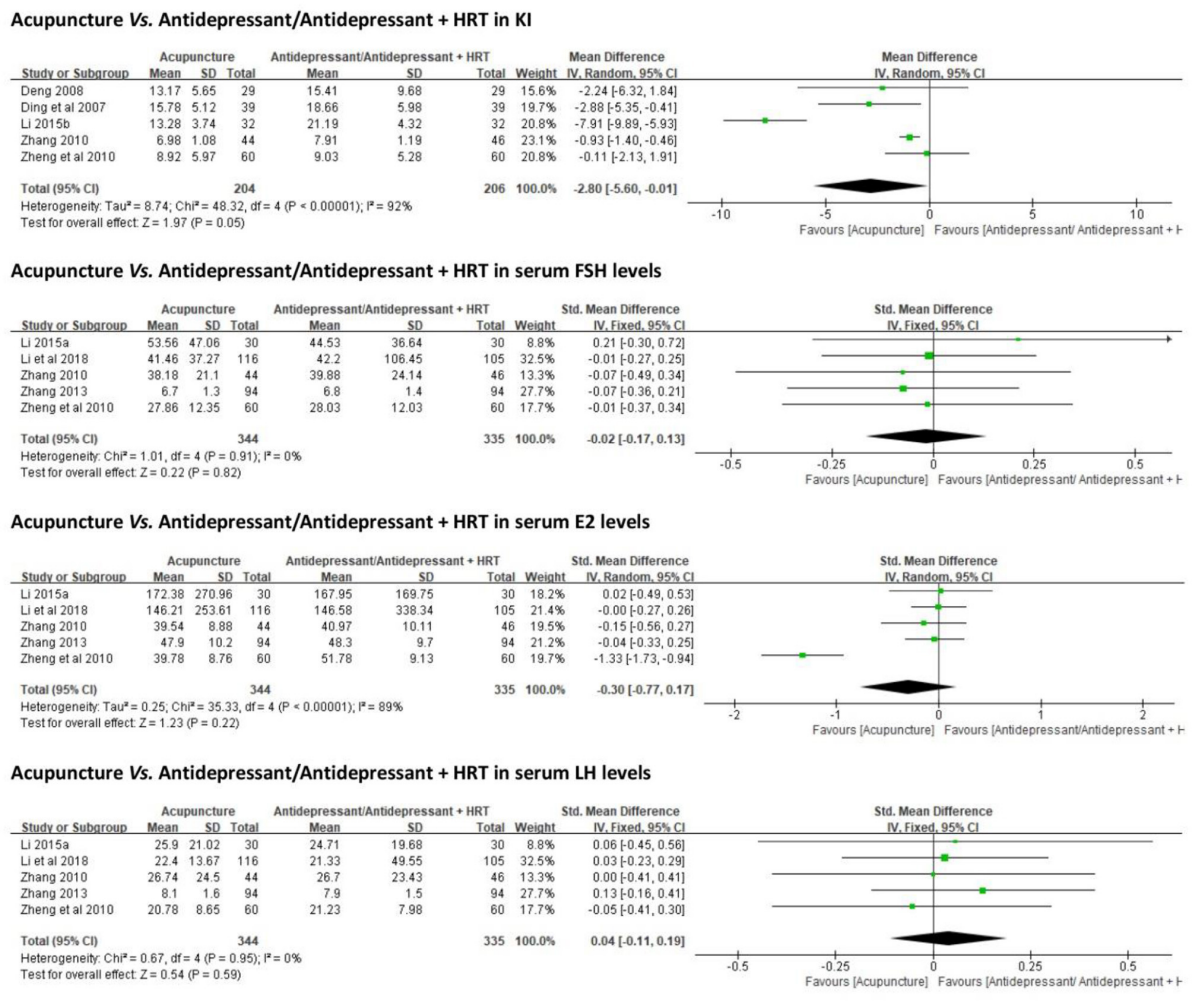
Forest plots of acupuncture vs. antidepressant/antidepressant + HRT in KI and serum hormone levels. HRT, hormone replacement therapy; KI, Kupperman index.

Five trials (38, 40, 48, 50, 51) (*n* = 275) employed KI as an outcome measure. No significant differences were identified between acupuncture and antidepressant/antidepressant + HRT in reducing KI scores [MD = −2.80, 95% CI (−5.60, −0.01), *p* = 0.05] (Figure 4).

Serum hormonal levels (FSH, E2, and LH) were reported in five studies (36, 37, 48–50) with contradictory results. When all data were combined, no statistically significant differences were identified between acupuncture and antidepressant/antidepressant + HRT in regulating FSH [SMD = −0.02, 95% CI (−0.17, 0.13), *p* = 0.82], E2 [SMD = −0.30, 95% CI (−0.77, 0.17), *p* = 0.22], or LH [SMD = 0.04, 95% CI (−0.11, 0.19), *p* = 0.59] (Figure 4).

##### Subgroup Analysis

Based on different acupuncture methods (MA or EA), or different standard care in control groups (antidepressant alone or antidepressant + HRT), we conducted subgroup analyses on HAMD (at post-treatment), HAMD (at 4-week follow-up), and KI scores, as well as serum E2 levels. However, the heterogeneity could not be fully explained. No interaction was identified in any subgroup. There was an interesting discovery about KI. When all five RCTs were pooled for analysis, there was no significant difference between acupuncture and standard care in reducing KI scores [MD = −2.80, 95% CI (−5.60, −0.01), *p* = 0.05]. However, in subgroup analysis, acupuncture showed better effects in decreasing KI scores, whether compared with antidepressant alone [MD = −4.55, 95% CI (−8.46, −0.65), *p* = 0.02] or antidepressant combined with HRT [MD = −0.89, 95% CI (−1.34, −0.43), *p* < 0.01]. We could not conduct subgroup analysis based on HAMD version, as three of 21 trials did not report which version of HAMD was used for assessment (Appendix 7).

##### Sensitivity Analysis

In an attempt to address the high heterogeneity, sensitivity analysis was performed based on the outcome of HAMD (at post-treatment) to ensure the results were not due to one or two studies. We chose influence analysis, by removing one study at a time and recalculating the combined estimate on the remaining studies to evaluate the stability of the results. We did not perform sensitivity analysis for the other outcome measures because of the small number of studies (<10).

**Figure 5:**
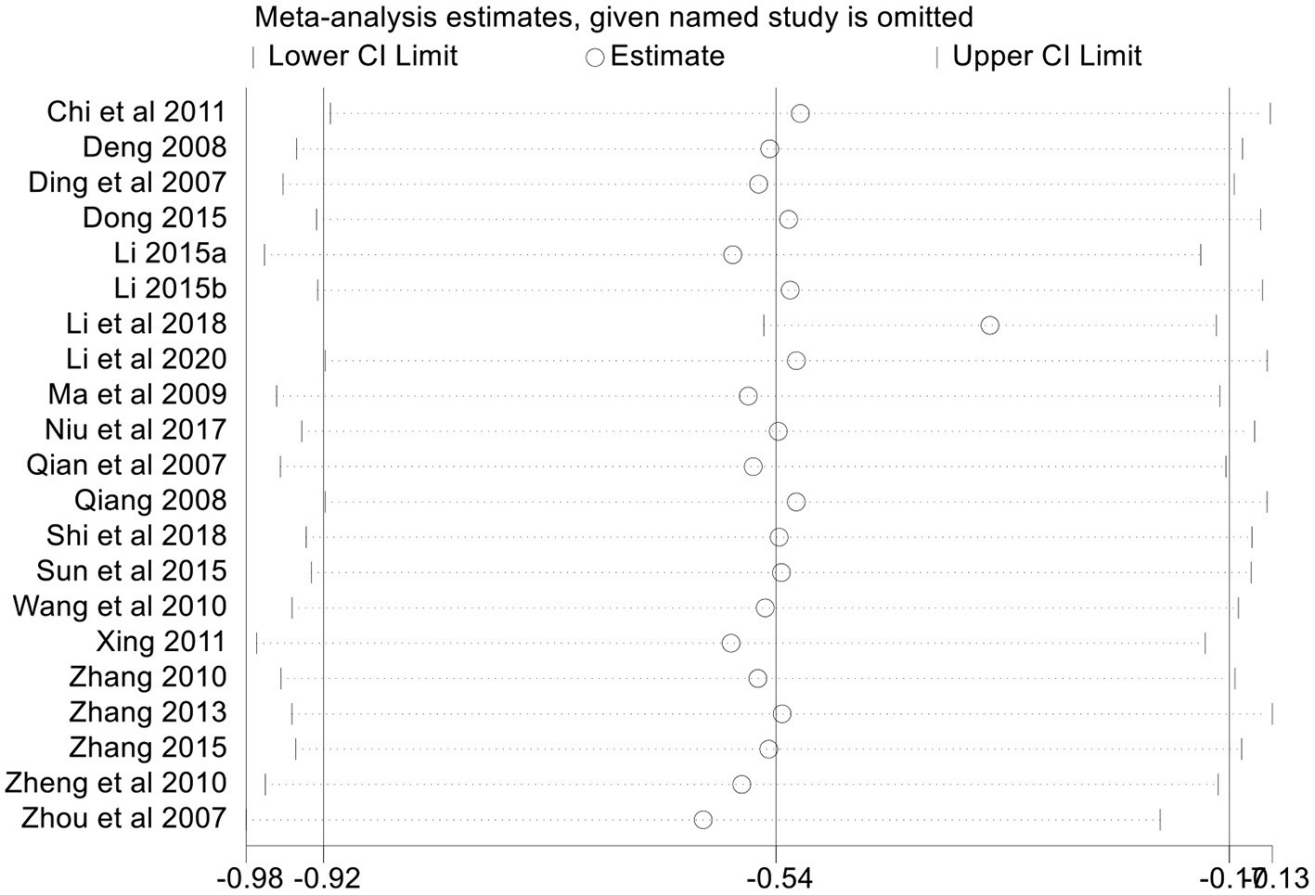
Sensitivity analysis based on HAMD. HAMD, Hamilton Depression Scale.

The results indicated that except for one study (37), each single study had little impact on the pooled estimate effects of HAMD, and the overall robustness and reliability of our study results were relatively high (Figure 5). That study (37) was thereby removed, and pooled estimate effects were recalculated. However, there were no significant changes in forest plots, and results still favored acupuncture in reducing HAMD global scores [SMD = −0.36, 95% CI (−0.54, −0.17), *p* < 0.01]. Heterogeneity was not completely explained with *I*^2^ only decreased from 93 to 70% (Appendix 8). These findings suggested that the study did not fully explain the heterogeneity. It may be one of the sources of heterogeneity.

##### Meta-Regression Analysis

Using HAMD (at post-treatment) as the outcome measure, we conducted univariate meta-regressions to investigate the sources of heterogeneity by treating publication year, study sample size, acupuncture stimulation (MA or EA), and standard care in control groups (antidepressant alone or antidepressant + HRT) as covariates. However, the heterogeneity across the 21 included studies could not be substantially explained by publication year (*I*^2^ = 91.02%, Tau2 = 0.54, *p* = 0.02), study sample size (*I*^2^ = 93.36%, Tau2 = 0.63, *p* = 0.10), acupuncture stimulation (*I*^2^ = 92.25%, Tau2 = 0.63, *p* = 0.12), and standard care in control groups (*I*^2^ = 93.47%, Tau2 = 0.72, *p* = 0.73) (Appendix 9, Supplementary Figures 1–4).

#### Acupuncture Combined With Antidepressant/Antidepressant + HRT vs. Antidepressant/Antidepressant + HRT

Three trials (55–57) were included (n = 275). Meta-analysis was only carried out for HAMD but not for other outcomes because there were fewer than three included trials for each of them.

##### Depression Symptoms

**Figure 6:**
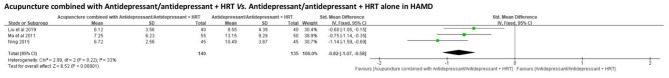
Forest plots of acupuncture combined with antidepressant/antidepressant + HRT vs. antidepressant/antidepressant + HRT in HAMD. HRT, hormone replacement therapy; HAMD, Hamilton Depression Scale.

HAMD was employed as an outcome in all three trials. The results favored acupuncture combined with antidepressant/antidepressant + HRT [SMD = −0.82, 95% CI (−1.07, −0.58), *p* < 0.01] (Figure 6).

##### Perimenopausal Symptoms and Hormonal Levels

None of the trials included an outcome related to perimenopausal symptoms, which hinder the judgment of difference between standard care alone and standard care combined with acupuncture in improving perimenopausal symptoms. However, one (56) of the three trials investigated the sex hormone levels of patients at pre- and post-treatment and found that standard care combined with acupuncture was more effective in down-regulating FSH levels and up-regulating E2 levels.

#### Acupuncture vs. Waitlist Control

No studies were identified under this comparison.

### Publication Bias Test

**Figure 7:**
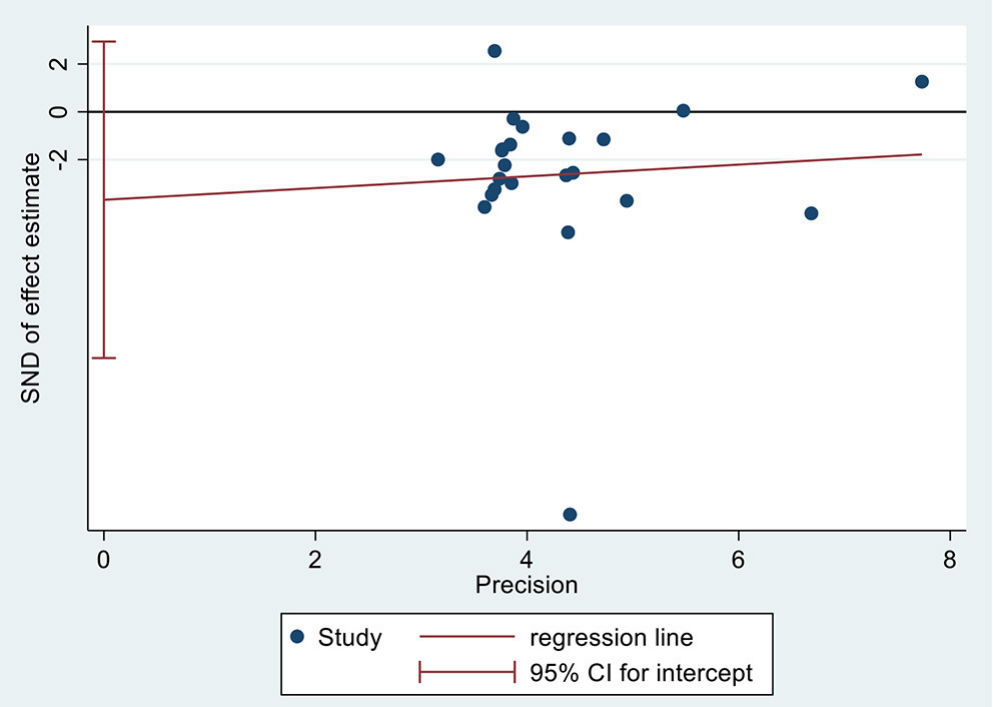
Publication bias test based on HAMD. HAMD, Hamilton Depression Scale.

We used linear regression analysis (Egger's test) to detect the publication bias based on HAMD in 25 included studies, and we found no statistically significant effect (*p* = 0.261) (Figure 7). Publication bias tests were not conducted for the other outcome measures because of the small number of studies (<10).

## Discussion

### Summary of Findings

Acupuncture appears to have better effects in reducing PMD than sham acupuncture. Acupuncture alone or combined with standard care (antidepressant/antidepressant + HRT) is superior to standard care alone in improving depressed mood in perimenopausal women. The reduction of HAMD global score varied from 1.4 to 3.6 points, and the reduction is of clinical relevance. Acupuncture showed better effects than or equivalent effects to antidepressants (escitalopram or Deanxit) in decreasing HAMD global scores at 2-, 4-, and 12-week follow-ups, suggesting that acupuncture may have intermediate- and long-term therapeutic effects on PMD, and its short-term effect was superior to antidepressants. Not enough data were reported on whether acupuncture also has intermediate- and long-term effects on perimenopausal symptoms other than depression in women with PMD. Whether or not the benefits of acupuncture were mediated *via* regulating serum hormone levels, such as FSH, E2, and LH, remains unclear because there were insufficient data. Not enough studies reported if acupuncture could reduce the side effects of HRT or antidepressants, as only one study (55) with a small sample size addressed this comparison. Acupuncture appeared to be well-tolerated and safe, as the AEs were only mild and far less than those for standard care. The most frequent AE was hematoma, which usually healed quickly after the needles were removed. Overall, the quality of the studies was low to moderate due to a lack of blinding of patients and outcome assessors.

### Strengths, Limitations, and Comparison With Previous Systematic Reviews

To the best of our knowledge, this was the first systematic review and meta-analysis comprehensively investigating if acupuncture can be recommended as an independent or adjuvant management to standard care for PMD. Women in Western countries are not likely to immediately give up Western medicine and choose acupuncture. However, they may be more willing to adopt acupuncture as adjuvant therapy to Western medication as part of a comprehensive management program (58, 59). Our review specifically addresses this question and supports a better effect of acupuncture alone or when combined with standard care.

We are aware of five previous systematic reviews (two in Chinese and three in English) that addressed a similar topic (26–29, 60). However, three of them were carried out more than 5 years ago (27–29). Three of the five reviews included many different forms of acupoint-based therapies, such as moxibustion (28, 29, 60), intradermal needling (28), acupoint catgut implantation (60), and/or even psychotherapy (60) and Chinese herbal medicine (60). Such practice introduces extra variability and makes it difficult to interpret the results. We only focused on common forms of acupuncture (MA or EA) to reduce variability and to better reflect the real clinical practice. It is worth mentioning that an incomplete retrieval issue was identified in one of the five reviews published in a peer-reviewed journal in 2020 (26). The review that reported no available RCT with acupuncture vs. placebo/sham acupuncture was retrieved (searching time: October 2018). However, two RCTs (35, 36) with this design (published in 2015) were identified and included in our review. Another issue with that review (26) was an RCT that was mistakenly included. The interventions of that review (26) were limited to only MA or EA like ours, but it included an RCT that used both acupuncture and moxibustion (61). Finally, these five reviews did not consider/mention the different versions of HAMD used in the included RCTs. MD but not SMD therefore was inappropriately used for pooling the estimated effect size, which reduced the reliability of their results.

In addition to the stricter selection criteria, usage of widely accepted analyses tools as mentioned above, the advantages of our review also included the following: (1) we conducted meta-analysis on HAMD at post-treatment as well as at 2-, 4-, and 12-week follow-ups; (2) we included the comparison of acupuncture + standard care vs. stand care alone and found acupuncture combined with standard care showed a better antidepressant effect than standard care alone, indicating that acupuncture can be considered as an adjuvant management in future treatment program; (3) we carried out meta-analysis and subgroup analysis for sex hormonal levels to elucidate the potential factors mediating the effect of acupuncture in perimenopausal women; (4) we employed STRICTA checklist to assess the reporting quality of acupuncture. These merits were not identified in any of previous systematic review.

This review has a few limitations. First, the meta-analysis was limited by the number of studies and small sample sizes despite our comprehensive search. Second, the quality of included studies was less than satisfactory. Third, the heterogeneity was high among the studies. We employed subgroup, sensitivity, and meta-regression analyses but could not identify the sources. Fourth, there were insufficient studies (<3) comparing acupuncture with placebo/sham acupuncture supporting a meta-analysis. Furthermore, there are potential flaws of design in these two RCTs (35, 36), which restricts us from more confidently recommending acupuncture as an independent remedy in the management of PMD. Fifth, some included studies did not clearly describe acupuncture details including depth of insertion and/or needle type used. Acupuncture is a complex intervention, and the skills of operators are important. However, only one study (37) explained the background of the trial acupuncturist. Those limitations impact the reproducibility and assessment of the real contribution. Finally, all the included RCTs were conducted in China. It is unknown if the results could be replicated in women outside of China. Further rigorous and well-designed RCTs with larger sample sizes and a multicenter design were required to build stronger evidence. The reporting quality of acupuncture should also be more detailed in order to improve the reproducibility of the treatment procedure as well as to facilitate the usage of this remedy by clinical practitioners.

Considering the consistency in findings and deficiency in study quality, we rate the strength of evidence being low to moderate, supporting the positive effect of acupuncture.

### Interpretation of Findings

Thousands of years ago, Chinese medical practitioners had realized the concept of perimenopause, and the etiology and pathogenesis of perimenopausal syndrome, as well as put forward the principles of diagnosis and treatment (e.g., herbal medicine and acupuncture) (62). Different from interpretation of hypothalamic–pituitary–gonadal axis in Western medicine, TCM believes perimenopausal disorders including PMD are caused by imbalance/disharmony of *Yin* and *Yang*, and *Zang-Fu*, which is expected to be balanced/harmonized with the intervention of acupuncture (62). Until now, acupuncture is still widely used in China to manage various physical and mental symptoms associated with menopause (63). Despite the promising results, the evidence quality of two included trials comparing acupuncture vs. sham acupuncture was poor (35, 36). Neither of these two studies carried out sample size calculation or intention-to-treat (ITT) analysis, which may partially weaken the reliability of the results. Sham acupuncture in these two trials used the same acupoints as those in the real-acupuncture group, with shallow insertion. Based on advanced medical imaging technology (laser Doppler blood-flow imaging), Huang et al. reported that deep or shallow acupuncture at acupoints caused decline in the ratio of blood-flow perfusion, while this phenomenon was not found in acupuncture at non-acupoints or in placebo-acupuncture (non-invasive) intervention (64), suggesting that deep or shallow needling on acupoints can trigger the desired physiological effects. Shallow acupuncture on acupoints is thereby not an appropriate placebo control (65). Future research should include effective sample size calculation, appropriate sham acupuncture control, which is near non-acupoints or acupoints unrelated to depression/menopausal symptoms with shallow needling and without *De-qi* sensation, an ITT analysis for outcomes, and more comprehensive follow-ups.

A three-point difference on HAMD is regarded as the “minimal improvement” (66). Our review found that acupuncture was better than standard care alone in reducing HAMD score by 1.4–3.6, which is of clinical significance—acupuncture is better than or at least equivalent to antidepressant in improving perimenopausal women's depression. In addition to the satisfactory short-term effects, the intermediate- and long-term benefits of acupuncture against PMD outlast those of antidepressants. Long-term clinical efficacy is crucial in the management of depressive symptoms, as depression is characterized by a high recurrence rate (67). Frequent relapse of depression (67) and withdrawal symptoms of antidepressants (68) are also two major reasons for numerous patients reject psychotropic agents and seek help from CAM therapy (36).

It is interesting to note that acupuncture also improved perimenopausal symptoms (decreased KI scores), better than either antidepressant alone or antidepressant combined with HRT, reflecting different underlying mechanisms of the two interventions (acupuncture vs. *p*harmacotherapy). A strong association between depression and changes in hormonal milieu has been widely established (69–71). Previous studies demonstrated that increased FSH and LH are linked to the depressed mood in women with no history of depression during their menopause transition (69); decreased E2 enhanced the risk for menopause-related depression and anxiety (70). Another study that reported contradictory results that PMD was associated with increased variability of E2 (69). Animal studies further explained the pathway on how hormonal fluctuations trigger the development of menopause-related depression. Gu et al. reported that increased FSH and LH contributed to the lower neurotransmitter release, such as 5-hydroxytryptamine (5-HT), norepinephrine (NE), and dopamine (DA), which might in turn cause the depression syndromes in menopause (71). Based on the PMD mouse model, Guo and colleagues reported EA significantly reduced mice's depressed performance, reflected by decreased time of forced swimming and tail suspension, increased number of spontaneous activities, etc. They also observed increased 5-HT, NE, and DA in mice's cerebral tissue, as well as increased serum E2 and decreased serum FSH and LH (72). However, in our review, the changes in those hormones in the acupuncture group did not differ from those in antidepressant/antidepressant + HRT group. Whether hormonal regulation mediates the effect of acupuncture on PMD thereby requires further investigation. For neurotransmitters, only one included study (56) reported that compared with sertraline, MA + sertraline was more effective in increasing serum 5-HT and γ-aminobutyric acid (GABA) levels, which are the major neurotransmitters involved in depression (73, 74). Therefore, this potential “cascade” phenomenon of acupuncture affecting sex hormones, which in turn affects neurotransmitter regulation, could be further investigated. In addition, KI score was also improved. KI measures both somatic and mental perimenopausal symptoms, including vasomotor symptoms, anxiety, and insomnia (75); all of these could contribute to depression in perimenopausal women (7).

The second aim of this systematic review was to investigate if acupuncture could further enhance the clinical efficacy and/or reduce the adverse reactions caused by these Western medications. While three RCTs (55–57) in this category showed that the combined therapy was more significantly effective in improving PMD than standard care, only one trial reported AEs in EA combined paroxetine was slightly less than paroxetine alone (2/55 vs. 3/50). Future studies are thereby needed to further explore the safety of a combined therapy of acupuncture and standard care for PMD.

According to the “Guidelines for the Evaluation and Treatment of Perimenopausal Depression” issued by Board of Trustees for the North American Menopause Society (NAMS), sleep disturbance, particularly insomnia, should be a part of PMD management (7). Depression comorbid insomnia is very common in perimenopausal women (76). However, we did not identify any study including the assessment for sleep. To understand the relationship between PMD and insomnia as well as the comprehensiveness of acupuncture's effects, future studies need to include validated sleep measures such as Pittsburgh Sleep Quality Index (PSQI), Insomnia Severity Index (ISI), or actigraphy for sleep assessment in women with PMD.

## Conclusions

This review has provided a low-to-moderate level of evidence supporting acupuncture as a safe and effective alternative to or adjuvant to standard care (antidepressant/antidepressant + HRT) in improving depressed mood as well as other menopause-related symptoms among women with PMD. Future studies need to include appropriate sham/placebo acupuncture and patient-assessor blinding methods in the trial designs, clarify whether acupuncture could also be an adjuvant to HRT, observe the intermediate- and long-term effects of acupuncture on perimenopausal symptoms other than depression in women with PMD, and understand if the improvement is associated with reproductive hormone changes induced by acupuncture. The assessment of sleep index should also be included.

## Data Availability Statement

The original contributions presented in the study are included in the article/Supplementary Material, further inquiries can be directed to the corresponding authors.

## Author Contributions

W-JZ and ZZ designed this review. Q-QF and F-YZ performed database search, data extraction, and statistical analyses. ZZ was involved in the quality assessment and bias risk analysis. F-YZ drafted the manuscript. GK, RC, and ZZ provided critical comments for revising the manuscript. All authors contributed to the article and approved the submitted version.

## Conflict of Interest

The authors declare that the research was conducted in the absence of any commercial or financial relationships that could be construed as a potential conflict of interest.
