# Supplementary material for: Acupuncture as an Independent or Adjuvant Management to Standard Care for Perimenopausal Depression: A Systematic Review and Meta-Analysis
Source: Front Psychiatry. 2021 May 28;12:666988. doi: 10.3389/fpsyt.2021.666988 (PMC8192720; doi:10.3389/fpsyt.2021.666988)

Appendix ____

Appendix 1. Search strategy

The following English search strategy was used for MEDLINE via PubMed while the Chinese one was used for SinoMed; both two search strategies were also suitable for other electronic databases:

Medline search strategy for English databases

No.	Search terms
1	acupuncture[MeSH Terms]
2	acupuncture
3	electropuncture
4	manual acupuncture
5	auricular acupuncture
6	acupuncture point
7	acupoint
8	#1 or #2 or #3 or #4 or #5 or #6 or #7
9	depression
10	depressive disorder
11	#9 or #10
12	climacteric
13	menopause
14	perimenopause
15	#12 or #13 or #14
16	#8 and #11 and #15

Medline search strategy for Chinese database

**No.**	**Search terms**
1	随机对照试验
2	随机
3	对照
4	(#3) OR (#2) OR (#1)
5	"针灸疗法"[不加权:扩展]
6	针灸
7	电针
8	穴
9	手捻针
10	针
11	(#10) OR (#9) OR (#8) OR (#7) OR (#6) OR (#5)
12	"抑郁症"[不加权:扩展]
13	抑郁
14	郁证
15	郁病
16	(#15) OR (#14) OR (#13) OR (#12)

17	"更年期"[不加权:扩展]
18	围绝经期
19	(#18) OR (#17)
20	(#19) AND (#16) AND (#11) AND (#4)

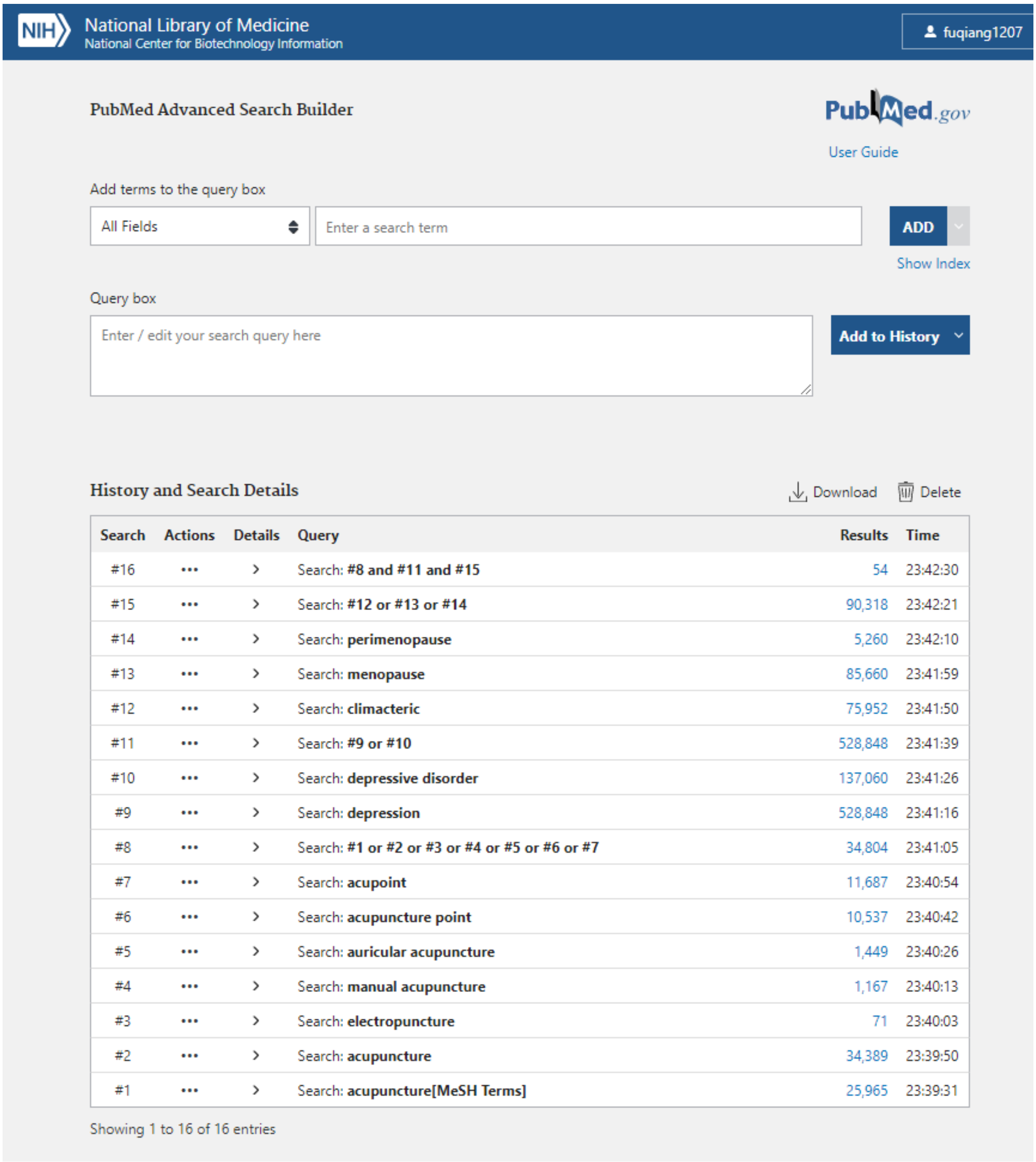
National Library of Medicine
National Center for Biotechnology Information
fuqiang1207

PubMed Advanced Search Builder

PubMed.gov

User Guide

Add terms to the query box

All Fields | Enter a search term | ADD

Show Index

Query box

Enter / edit your search query here

Add to History

History and Search Details

Download Delete

Search	Actions	Details	Query	Results	Time
#16	•••	>	Search: **#8 and #11 and #15**	54	23:42:30
#15	•••	>	Search: **#12 or #13 or #14**	90,318	23:42:21
#14	•••	>	Search: **perimenopause**	5,260	23:42:10
#13	•••	>	Search: **menopause**	85,660	23:41:59
#12	•••	>	Search: **climacteric**	75,952	23:41:50
#11	•••	>	Search: **#9 or #10**	528,848	23:41:39
#10	•••	>	Search: **depressive disorder**	137,060	23:41:26
#9	•••	>	Search: **depression**	528,848	23:41:16
#8	•••	>	Search: **#1 or #2 or #3 or #4 or #5 or #6 or #7**	34,804	23:41:05
#7	•••	>	Search: **acupoint**	11,687	23:40:54
#6	•••	>	Search: **acupuncture point**	10,537	23:40:42
#5	•••	>	Search: **auricular acupuncture**	1,449	23:40:26
#4	•••	>	Search: **manual acupuncture**	1,167	23:40:13
#3	•••	>	Search: **electropuncture**	71	23:40:03
#2	•••	>	Search: **acupuncture**	34,389	23:39:50
#1	•••	>	Search: **acupuncture[MeSH Terms]**	25,965	23:39:31

Showing 1 to 16 of 16 entries

Appendix 2. Valid outcome measures at different timepoint in each study

Author, year	Type of outcome measures	Pre-treatment	Post-treatment	Follow-up
Wang et al 2015 [35]	Depression	SDS	SDS	SDS
	Perimenopausal symptoms	MENQOL	MENQOL	MENQOL
Li 2015a [36]	Depression	HAMD	HAMD	HAMD
	Perimenopausal symptoms	MENQOL, FSH, E2, LH	MENQOL, FSH, E2, LH	MENQOL
Li et al 2018 [37]	Depression	HAMD	HAMD	HAMD
	Perimenopausal symptoms	MENQOL, FSH, E2, LH	MENQOL, FSH, E2, LH	MENQOL
Chi et al 2011 [30]	Depression	HAMD	HAMD	/
Deng 2008 [38]	Depression	HAMD	HAMD	HAMD
	Perimenopausal symptoms	KI	KI	KI
Dong 2015 [39]	Depression	HAMD	HAMD	/
Li 2015b [40]	Depression	HAMD	HAMD	HAMD
	Perimenopausal symptoms	KI	KI	/
Ma et al 2009 [41]	Depression	HAMD	HAMD	/
Niu et al 2017 [42]	Depression	HAMD	HAMD	/
Qian et al 2007 [43]	Depression	HAMD	HAMD	/
Qiang 2008 [44]	Depression	HAMD	HAMD	/
Shi et al 2018 [45]	Depression	HAMD	HAMD	HAMD
Sun et al 2015 [46]	Depression	HAMD	HAMD	/
Wang et al 2010 [47]	Depression	HAMD	HAMD	HAMD
Zhang 2010 [48]	Depression	HAMD	HAMD	/
	Perimenopausal symptoms	KI, FSH, E2, LH	KI, FSH, E2, LH	/
Zhang 2013 [49]	Depression	HAMD	HAMD	/
	Perimenopausal symptoms	FSH, E2, LH	FSH, E2, LH	/
Zheng et al 2010 [50]	Depression	HAMD	HAMD	HAMD
	Perimenopausal symptoms	KI, FSH, E2, LH	KI, FSH, E2, LH	KI
Ding et al 2007 [51]	Depression	HAMD	HAMD	/
	Perimenopausal symptoms	KI	KI	/
Li et al 2020 [52]	Depression	HAMD	HAMD	/
Zhang 2015 [53]	Depression	HAMD	HAMD	HAMD
Xing 2011 [54]	Depression	HAMD	HAMD	/
Zhou et al 2007 [31]	Depression	HAMD	HAMD	/
Ma et al 2011 [55]	Depression	HAMD	HAMD	/
Liu et al 2019 [56]	Depression	HAMD	HAMD	/
	Perimenopausal symptoms	FSH, E2	FSH, E2	/
Ning 2015 [57]	Depression	HAMD	HAMD	/

Abbreviations: HAMD, Hamilton Depression Scale; SDS, Self-Rating Depression Scale; KI, Kupperman Index; MENQOL, Menopause-Specific Quality of Life; FSH, follicle stimulating hormone; LH, luteinizing hormone; E2, estradiol

Appendix 3. Risk of bias graph

	Random sequence generation (selection bias)	Allocation concealment (selection bias)	Blinding of participants and personnel (performance bias)	Blinding of outcome assessment (detection bias)	Incomplete outcome data (attrition bias)	Selective reporting (reporting bias)	Other bias
Chi et al 2011	?	?	−	?	+	?	+
Deng 2008	+	?	−	?	+	?	+
Ding et al 2007	?	?	−	?	+	?	+
Dong 2015	?	?	−	?	+	?	+
Li 2015a	+	+	−	+	+	?	+
Li 2015b	?	?	−	?	+	?	+
Li et al 2018	+	+	−	+	+	+	+
Li et al 2020	+	?	−	?	+	?	+
Liu et al 2019	+	?	−	?	+	?	+
Ma et al 2009	?	?	−	?	+	?	+
Ma et al 2011	?	?	−	?	+	?	+
Ning 2015	+	?	−	?	+	?	+
Niu et al 2017	+	?	−	?	+	?	+
Qian et al 2007	+	?	−	?	+	?	+
Qiang 2008	+	?	−	?	+	?	+
Shi et al 2018	?	?	−	?	+	?	+
Sun et al 2015	?	?	−	?	+	?	+
Wang et al 2010	+	?	−	?	+	?	+
Wang et al 2015	+	?	−	?	+	?	+
Xing 2011	+	?	−	?	+	?	+
Zhang 2010	+	?	−	?	−	?	+
Zhang 2013	?	?	−	?	+	?	+
Zhang 2015	?	?	−	?	+	?	+
Zheng et al 2010	+	?	−	?	+	?	+
Zhou et al 2007	+	?	−	?	+	?	+

Notes: other bias are assessed based on baseline balance

Appendix 4. Methodological quality assessment of 25 included RCTs

Author, year	Random sequence generation	Allocation concealment	Blinding of participants	Blinding of personnel	Blinding of outcome assessment	Incomplete outcome data	Selective outcome reporting	Other bias (baseline balance)	Other bias (funding or conflict of interest)
Wang et al 2015 [35]	L	U	U	H	U	L	U	L	L
Li 2015a [36]	L	L	L	H	L	L	U	L	L
Li et al 2018 [37]	L	L	H	H	L	L	L	L	L
Chi et al 2011 [30]	U	U	H	H	U	L	U	L	U
Deng 2008 [38]	L	U	H	H	U	L	U	L	U
Dong 2015 [39]	U	U	H	H	U	L	U	L	L
Li 2015b [40]	U	U	H	H	U	L	U	L	U
Ma et al 2009 [41]	U	U	H	H	U	L	U	L	U
Niu et al 2017 [42]	L	U	H	H	U	L	U	L	U
Qian et al 2007 [43]	L	U	H	H	U	L	U	L	U
Qiang 2008 [44]	L	U	H	H	U	L	U	L	U
Shi et al 2018 [45]	U	U	H	H	U	L	U	L	L
Sun et al 2015 [46]	U	U	H	H	U	L	U	L	L
Wang et al 2010 [47]	L	U	H	H	U	L	U	L	L
Zhang 2010 [48]	L	U	H	H	U	H	U	L	U
Zhang 2013 [49]	U	U	H	H	U	L	U	L	U
Zheng et al 2010 [50]	L	U	H	H	U	L	U	L	U
Ding et al 2007 [51]	U	U	H	H	U	L	U	L	U
Li et al 2020 [52]	L	U	H	H	U	L	U	L	U
Zhang 2015 [53]	U	U	H	H	U	L	U	L	U
Xing 2011 [54]	L	U	H	H	U	L	U	L	U

Zhou et al 2007 [31]	L	U	H	H	U	L	U	L	L
Ma et al 2011 [55]	U	U	H	H	U	L	U	L	U
Liu et al 2019 [56]	L	U	H	H	U	L	U	L	U
Ning 2015 [57]	L	U	H	H	U	L	U	L	U

Abbreviations: L, Low risk; U, Unclear risk; H, High risk.

Appendix 5. Details of acupuncture procedure based on revised STRICTA (2010 Version)

Item of STRICTA	*1. Acupuncture rationale*			*2. Needling details*							*3. Treatment regimen*		*4. Other components of treatment*		*5. Practitioner background*	6. Control or comparator interventions	
	(1a) Style of acupuncture	(1b) Rationale for treatment	(1c) Extent to which treatment was varied	(2a) Number of needles inserted	(2b) points used	(2c) Depths of insertion	(2d) Responses elicited	(2e) Needle stimulation	(2f) Needle retention time	(2g) Needle type	(3a) Number of treatment sessions	(3b) Frequency and duration of treatment sessions	(4a) Details of other interventions administered to the acupuncture group	(4b) Setting and context of treatment	Description of acupuncturist	(6a) Rationale for the control or comparator	(6b)Precise description of the control or comparator
Studies eligible to the items	[30,31,35,36,37,38,39,40,41,42,43,44,45,46,47,48,49,50,51,52,53,54,55,56,57]	[30,31,35,36,37,38,39,40,41,42,43,44,45,46,47,48,49,50,51,52,53,54,55,56,57]	/	[30,31,35,36,37,38,39,40,41,42,43,44,45,46,47,48,49,50,51,52,53,54,55,56,57]	[30,31,35,36,37,38,39,40,41,42,43,44,45,46,47,48,49,50,51,52,53,54,55,56,57]	[30,31,36,40,41,42,43,44,45,46,49,50,51,52,54,57]	[30,31,35,36,37,38,39,40,41,42,43,44,45,46,47,48,49,50,51,52,53,54,55,56,57]	[30,31,35,36,37,38,39,40,41,42,43,44,45,46,47,48,49,50,51,52,53,54,55,56,57]	[30,31,35,36,37,38,39,40,41,42,43,44,45,46,47,48,49,50,51,52,53,54,55,56,57]	[30,31,36,37,38,39,40,41,42,43,44,45,46,47,48,50,51,52,54,56,57]	[30,31,35,36,37,38,39,40,41,42,43,44,45,46,47,48,49,50,51,52,53,54,55,56,57]	[30,31,35,36,37,38,39,40,41,42,43,44,45,46,47,48,49,50,51,52,53,54,55,56,57]	[55,56,57]	/	[38]	[30,31,35,36,37,38,39,40,41,42,43,44,45,46,47,48,49,50,51,52,53,54,55,56,57]	[30,31,35,36,37,38,39,40,41,42,43,44,45,46,47,48,49,50,51,52,53,54,55,56,57]
Total number and percentage [n (%)]	25 (100)	25 (100)	0 (0)	25 (100)	25 (100)	16 (64)	25 (100)	25 (100)	25 (100)	21 (84)	25 (100)	25 (100)	3 (12)	0 (0)	1 (4)	25 (100)	25 (100)
Wang et al 2015 [35]	Chinese Acup	TCM theory	NR	Reported	KI6, LU7, PC6, SP4	NR	*De-qi*	MA	30 min	NR	8 weeks	30 min/day, 3 days/week	NR	NR	NR	Sham-MA	Reported (details in Table 1)
Li 2015a [36]	Chinese Acup	TCM theory	NR	Reported	CV4, EX-CA1, EX-HN3, GV20, LI4, LR3, SP6, ST25	0.5-1 *cun*	*De-qi*	EA	30 min	stainless steel (0.3*25 mm for	12 weeks	30 min/day, 3 days/week	NR	NR	NR	Sham-EA, Escitalopram	Reported (details in Table 1)

										EX-HN3 and GV20; 0.3*40 mm for LI4, LR3 and SP6,; 0.3*50 mm for CV4 and EX-CA1; 0.3*75 mm for ST25)							
Li et al 2018 [37]	Chinese Acup	TCM theory	NR	Reported	CV4, EX-CA1, EX-HN3, GV20, LI4, LR3, SP6, ST25	NR	*De-qi*	EA	30 min	stainless steel (0.3*25 mm, 0.3*40 mm and 0.3*50 mm)	12 weeks	30 min/day, 3 days/week	NR	NR	acupuncture experience > 5 years	Escitalopram	Reported (details in Table 1)
Chi et al 2011 [30]	Chinese Acup	TCM theory	NR	Reported	EX-HN1, EX-HN3, GV20, KI3, LR3, LR14, SP6, ST36	0.5-1 *cun*	*De-qi*	MA	30 min	stainless steel (0.35*40 mm)	4 weeks	30 min/day	NR	NR	NR	Fluoxetine	Reported (details in Table 1)
Deng 2008 [38]	Chinese Acup	TCM theory	NR	Reported	CV3, CV4, CV6, CV10, CV12, KI17, Qipang (0.5 Cun beside CV6), Xiafengshidian (1 Cun below and beside ST26)	NR	*De-qi*	MA	20-30 min	stainless steel (0.22*30 mm and 0.22*40 mm)	4 weeks	20-30 min/day, after 3 consecutive days of treatment, once treatment every 3 days	NR	NR	NR	Deanxit	Reported (details in Table 1)
Dong 2015 [39]	Chinese Acup	TCM theory	NR	Reported	BL13, BL15, BL17, BL18, BL20, BL21, BL23	NR	*De-qi*	MA	30 min	stainless steel (0.3*50 mm)	30 days	30 min/day	NR	NR	NR	Nilestriol + Fluoxetine	Reported (details in Table 1)
Li 2015b [40]	Chinese Acup	TCM theory	NR	Reported	BL15, BL18, BL23, EX-HN1, EX-HN3, GV20, GV24, PC6	0.5-1 *cun*	*De-qi*	MA	30 min	stainless steel (0.3*25 mm, 0.3*40 mm and 0.3*50 mm)	12 weeks	30 min/day, 6 days/week	NR	NR	NR	Fluoxetine	Reported (details in Table 1)
Ma et al 2009 [41]	Chinese Acup	TCM theory	NR	Reported	EX-HN1, EX-HN3, GV20, HT7, PC6, PC7, SP6, ST36	0.5-1 *cun*	*De-qi*	MA	30 min	stainless steel (0.35*40 mm)	8 weeks	30 min/day, 5 days/week	NR	NR	NR	Fluoxetine	Reported (details in Table 1)

Niu et al 2017 [42]	Chinese Acup	TCM theory	NR	Reported	BL13, BL15, BL17, BL18, BL20, BL23	17mm-27mm	De-qi	MA	30 min	stainless steel (0.25*25 mm for BL13, BL15, BL17, BL18, BL20; 0.25*40 mm for BL23)	6 weeks	30 min/day, 5 days/week	NR	NR	NR	Fluoxetine	Reported (details in Table 1)
Qian et al 2007 [43]	Chinese Acup	TCM theory	NR	Reported	BL13, BL15, BL17, BL18, BL20, BL23	0.5-0.8 *cun*	*De-qi*	MA	25 min	stainless steel (0.35*25 mm)	6 weeks	25 min/day, 5 days/week	NR	NR	NR	Fluoxetine	Reported (details in Table 1)
Qiang 2008 [44]	Chinese Acup	TCM theory	NR	Reported	BL15, BL18, BL23,EX-HN1, GB20	0.5-0.8 *cun*	*De-qi*	MA	25 min	stainless steel (0.35*25 mm)	6 weeks	25 min/day, 5 days/week	NR	NR	NR	Fluoxetine	Reported (details in Table 1)
Shi et al 2018 [45]	Chinese Acup	TCM theory	NR	Reported	CV4, EX-CA1, EX-HN3, GV20, LI4, LR3, SP6, ST25	0.5-1 *cun*	*De-qi*	EA	30 min	stainless steel (0.3*25 mm for EX-HN3 and GV20; 0.3*40 mm for LI4, LR3 and SP6,; 0.3*50 mm for CV4 and EX-CA1; 0.3*75 mm for ST25)	12 weeks	30 min/day, 3 days/week	NR	NR	NR	Escitalopram	Reported (details in Table 1)
Sun et al 2015 [46]	Chinese Acup	TCM theory	NR	Reported	CV4, EX-CA1, EX-HN3, GV20, LI4, LR3, SP6, ST25	0.5-1 *cun*	*De-qi*	EA	30 min	stainless steel (0.3*25 mm for EX-HN3 and GV20; 0.3*40 mm for LI4, LR3 and SP6,; 0.3*50 mm for CV4 and EX-CA1; 0.3*75 mm for ST25)	12 weeks	30 min/day, 3 days/week	NR	NR	NR	Escitalopram	Reported (details in Table 1)
Wang et al	Chinese Acup	TCM theory	NR	Reported	CV3, CV4, CV6, CV10,	NR	*De-qi*	MA	30 min	stainless steel	4 weeks	30 min/day, after	NR	NR	NR	Deanxit	Reported (details

2010 [47]					CV12, KI17					(0.22*30 mm and 0.22*40 mm)		3 consecutive days of treatment, once treatment every 3 days					in Table 1)
Zhang 2010 [48]	Chinese Acup	TCM theory	NR	Reported	BL13, BL15, BL17, BL20, BL23, GV20, KI3, LR3, PC6, SP6	NR	*De-qi*	EA	30 min	stainless steel (0.25*40 mm)	12 weeks	30 min/day, 5 days/week	NR	NR	NR	Nilestriol+ Fluoxetine	Reported (details in Table 1)
Zhang 2013 [49]	Chinese Acup	TCM theory	NR	Reported	EX-HN1, GB13, GV20, GV24, HT7	13-25mm	*De-qi*	MA	30 min	NR	12 weeks	30 min/day, 7 days/week	NR	NR	NR	Premarin + Provera + Fluoxetine	Reported (details in Table 1)
Zheng et al 2010 [50]	Chinese Acup	TCM theory	NR	Reported	BL8, BL18, BL23, GV19, GV21, KI3, LR3, SP6	1.5 *cun*	*De-qi*	MA	30 min (needle retaining time for 8 hour in BL8, GV19, GV21 per session)	stainless steel (0.3*50 mm)	12 weeks	30 min/day, 7 days/week	NR	NR	NR	Premarin + Provera + Fluoxetine	Reported (details in Table 1)
Ding et al 2007 [51]	Chinese Acup	TCM theory	NR	Reported	BL15, BL18, BL20, BL23, GV20, HT7, LR3, SP6	0.5-1.5 *cun*	*De-qi*	MA	30 min	stainless steel (0.35*40 mm)	4 weeks	30 min/day, 6 days/week	NR	NR	NR	Fluoxetine	Reported (details in Table 1)
Li et al 2020 [52]	Chinese Acup	TCM theory	NR	Reported	EX-HN1, EX-HN3,GV20, HT7, LI4, PC6, SP6, ST36	0.3-0.5 *cun*	*De-qi*	EA	25 min	stainless steel (0.25*40 mm)	6 weeks	25 min/day, 3 days/week	NR	NR	NR	Fluoxetine	Reported (details in Table 1)
Zhang 2015 [53]	Chinese Acup	TCM theory	NR	Reported	CV3, CV4, CV6, CV10, CV12, KI17	NR	*De-qi*	MA	30 min	NR	4 weeks	30 min/day, after 3 consecutive days of treatment, once	NR	NR	NR	Deanxit	Reported (details in Table 1)

												treatment every 3 days					
Xing 2011 [54]	Chinese Acup	TCM theory	NR	Reported	GV26, PC5	0.5 *cun*	*De-qi*	MA	20 min	stainless steel (0.35*25 mm)	6 weeks	20 min/day, 7 days/week	NR	NR	NR	Fluoxetine	Reported (details in Table 1)
Zhou et al 2007 [31]	Chinese Acup	TCM theory	NR	Reported	BL15, BL18, BL23, EX-HN1, GB13, GV24, SP6, ST36	18-30mm	*De-qi*	MA	30 min	stainless steel (0.3*25 mm and 0.3*40 mm)	6 weeks	30 min/day, 6 days/week	NR	NR	NR	Fluoxetine	Reported (details in Table 1)
Ma et al 2011 [55]	Chinese Acup	TCM theory	NR	Reported	EX-HN3, GV20, LI4, PC6, ST36	NR	*De-qi*	EA	45 min	NR	6 weeks	45 min/day, 7 days/week	NR	NR	NR	Paroxetine	Reported (details in Table 1)
Liu et al 2019 [56]	Chinese Acup	TCM theory	NR	Reported	BL23, CV4, HT7, KI3, LI4, LR3, SP6	NR	*De-qi*	MA	30 min	stainless steel (0.3*40 mm)	12 weeks	30 min/day, 3 days/week	NR	NR	NR	Sertraline	Reported (details in Table 1)
Ning 2015 [57]	Chinese Acup	TCM theory	NR	Reported	BL13, BL15, BL18, BL20, BL23, GV20, HT7, KI3, LI4, LR3	0.5-1.2 *cun*	*De-qi*	MA	30 min	stainless steel (0.3*40 mm)	12 weeks	30 min/day, 7 days/week	NR	NR	NR	Nilestriol + Fluoxetine	Reported (details in Table 1)

Abbreviations: NR, no record; Acup, acupuncture; TCM, Traditional Chinese Medicine; MA, manual acupuncture; EA, electroacupuncture; BL8, Luoque; BL13, Feishu; BL15, Xinshu; BL17, Geshu; BL18, Ganshu; BL20, Pishu; BL21, Weishu; BL23, Shenshu; CV3, Zhongji; CV4, Guanyuan; CV6, Qihai, CV10, Xiawan; CV12, Zhongwan; EX-CA1, Zigong; EX-HN1, Sishencong; EX-HN3, Yintang; GB13, Benshen; GV19, Houding; GV20, Baihui; GV21, Qianding; GV24, Shenting; GV26, Shuigou; HT7, Shenmen; KI6, Zhaohai; KI3, Taixi; KI13, Qixue; KI17, Shangqu; LI4, Hegu; LR3, Taichong; LR14, LU7, Lieque; Qimen; PC5, Jianshi; PC6, Neiguan; PC7, Daling; SP4, Gongsun; SP6, Sanyingjiao; ST25, Tianshu; ST26, Wailing; ST36, Zusanli; *De-qi* (obtaining Qi) refers to acupuncture-evoked specific sensations such as soreness, numbness, heaviness, and distention at the site of needle placement, and these sensations may spread to other parts of the body

Appendix 6. Qualitative and quantitative analysis in the 25 included studies

Interventions *VS.* controls	Number of studies	Qualitative analysis	Quantitative analysis				
			pooled effect sizes	subgroup analysis	sensitivity analysis	meta-regression analysis	Publication bias
Acup. *VS.* sham Acup.	2	All	/	/	/	/	HAMD: 24 studies (one out of 25 studies used SDS instead of HAMD)
Acup. *VS.* antidepressant or antidepressant combined with HRT	21	All	(i) HAMD: 21 studies<br>(ii) HAMD (2-week follow-up): 3 studies<br>(iii) HAMD (4-week follow-up): 6 studies<br>(iv) HAMD (12-week follow-up): 3 studies<br>(v) KI: 5 studies<br>(vi) FSH: 5 studies<br>(vii) E2: 5 studies<br>(viii) LH: 5 studies	HAMD	HAMD	HAMD	
acupuncture + antidepressant or antidepressant combined with HRT *VS.* antidepressant or antidepressant combined with HRT	3	All	(i) HAMD: 3 studies	/	/	/	

Abbreviations: Acup., acupuncture; HRT, hormone replacement therapy; HAMD, Hamilton Depression Scale; KI, Kupperman index; FSH, follicle stimulating hormone; LH, Luteinizing hormone; E2, estradiol

Appendix 7. Subgroup analyses of HAMD and KI (Acupuncture *Vs.* Antidepressant/Antidepressant + HRT)

Basis for subgroup classification	All trials or subgroup title	No. of Studies	No. of participants	Statistical method	Effect size	*p*	I^2	Subgroup analysis results
HAMD at post-treatment	All trials	21	1842	Std. Mean Difference (IV, Random, 95% CI)	-0.54 [-0.91, -0.16]	< 0.01	93	
Acupuncture stimulations	(i) MA	15	1309	Std. Mean Difference (IV< random, 95% CI)	-0.34 [-0.56, -0.12]	< 0.01	73	Chi^2 statistic 1.00, df = 1, *p* = 0.32
	(ii) EA	6	533	Std. Mean Difference (IV< random, 95% CI)	-0.99 [-2.24, -0.26]	0.12	97	
Type of standard care	(i) *Vs.* antidepressant alone	17	1384	Std. Mean Difference (IV, Random, 95% CI)	-0.57 [-1.05, -0.09]	0.02	94	Chi^2 statistic 0.35, df = 1, *p* = 0.55
	(ii) *Vs.* antidepressant + HRT	4	458	Std. Mean Difference (IV< random, 95% CI)	-0.39 [-0.73, -0.04]	0.03	69	
HAMD at 4-week follow-up	All trials	6	504	Std. Mean Difference (IV< random, 95% CI)	-1.36 [-2.72, 0.00]	0.05	97	
Acupuncture stimulations	(i) MA	3	163	Std. Mean Difference (IV, Random, 95% CI)	-0.82 [-1.14, -0.49]	< 0.01	0	Chi^2 statistic 0.60, df = 1, *p* = 0.44
	(ii) EA	3	341	Std. Mean Difference (IV< random, 95% CI)	-1.91 [-4.63, 0.82]	0.17	99	
KI	All trials	5	410	Mean Difference (IV, Random, 95% CI)	-2.80 [-5.60, -0.01]	0.05	90	
Acupuncture stimulations	(i) MA	4	320	Mean Difference (IV< random, 95% CI)	-3.34 [-7.25, 0.57]	0.09	/	Chi^2 statistic 1.44, df = 1, *p* = 0.23
	(ii) EA	1	90	Mean Difference (IV< random, 95% CI)	-0.93 [-1.40, -0.46]	< 0.01	92	

Type of standard care	(i) *Vs.* antidepressant alone	3	200	Mean Difference (IV, Random, 95% CI)	-4.55 [-8.46, -0.65]	0.02	84	Chi^2 statistic 3.34, df = 1, p< 0.07
	(ii) *Vs.* antidepressant + HRT	2	210	Mean Difference (IV, Random, 95% CI)	-0.89 [-1.34, -0.43]	< 0.01	0	
Serum E2 levels	All trials	5	679	Std. Mean Difference (IV, Random, 95% CI)	-0.30 [-0.77, 0.17]	0.22	89	
Acupuncture stimulations	(i) MA	2	308	Std. Mean Difference (IV, Random, 95% CI)	-0.68 [-1.95, 0.59]	0.29	96	Chi^2 statistic 0.97, df = 1, p = 0.32
	(ii) EA	3	371	Std. Mean Difference (IV, Random, 95% CI)	-0.03 [-0.24, 0.17]	0.75	0	
Type of standard care	(i) *Vs.* antidepressant alone	2	281	Std. Mean Difference (IV, Random, 95% CI)	0.00 [-0.23, 0.24]	0.98	0	Chi^2 statistic 1.43, df = 1, p = 0.23
	(ii) *Vs.* antidepressant + HRT	3	398	Std. Mean Difference (IV, Random, 95% CI)	-0.50 [-1.30, 0.29]	0.22	93	

Abbreviations: MA, manual acupuncture; EA, electroacupuncture

Appendix 8. Forest plot after removing a study that may be the potential source of heterogeneity

	Acupuncture			Antidepressant/Antidepressant + HRT				Std. Mean Difference
Study or Subgroup	Mean	SD	Total	Mean	SD	Total	Weight	IV, Random, 95% CI
Chi et al 2011	11.52	5.35	30	16.3	4.72	30	4.6%	-0.94 [-1.47, -0.40]
Deng 2008	8.1	3.53	29	10.31	6.33	29	4.7%	-0.43 [-0.95, 0.10]
Ding et al 2007	14.73	3.32	39	15.66	3.98	39	5.2%	-0.25 [-0.70, 0.19]
Dong 2015	11.73	5.58	30	15.32	3.86	30	4.7%	-0.74 [-1.26, -0.21]
Li 2015a	9.33	3	30	8.75	3.27	30	4.8%	0.18 [-0.32, 0.69]
Li 2015b	9.41	4.78	32	13.53	5.81	32	4.8%	-0.77 [-1.27, -0.26]
Li et al 2020	7.2	3.3	30	9.7	2.3	30	4.6%	-0.87 [-1.40, -0.34]
Ma et al 2009	8.1	5.07	30	8.52	6.15	30	4.8%	-0.07 [-0.58, 0.43]
Niu et al 2017	7.7	4.3	41	10.2	4.4	41	5.2%	-0.57 [-1.01, -0.13]
Qian et al 2007	10.58	4.18	33	11.24	4.15	30	4.9%	-0.16 [-0.65, 0.34]
Qiang 2008	7.2	3.5	30	9.8	2.3	30	4.6%	-0.87 [-1.40, -0.34]
Shi et al 2018	8.5	4.32	30	10.9	3.81	30	4.7%	-0.58 [-1.10, -0.06]
Sun et al 2015	6.57	2.84	21	8.86	4.27	21	4.1%	-0.62 [-1.24, 0.00]
Wang et al 2010	8.6	3.9	30	10.6	6.9	30	4.8%	-0.35 [-0.86, 0.16]
Xing 2011	8.86	4.01	120	8.17	4.44	120	6.6%	0.16 [-0.09, 0.42]
Zhang 2010	7.42	2.8	44	8.02	2.08	46	5.4%	-0.24 [-0.66, 0.17]
Zhang 2013	12.3	3.1	94	14.4	3.5	94	6.3%	-0.63 [-0.93, -0.34]
Zhang 2015	9.5	3.7	29	11	3.5	29	4.7%	-0.41 [-0.93, 0.11]
Zheng et al 2010	10.02	4.21	60	9.98	4.78	60	5.8%	0.01 [-0.35, 0.37]
Zhou et al 2007	8.9	4.06	30	6.36	3.23	28	4.6%	0.68 [0.15, 1.21]
**Total (95% CI)**			**812**			**809**	**100.0%**	**-0.36 [-0.54, -0.17]**

Heterogeneity: $Tau^2 = 0.12$; $Chi^2 = 62.47$, $df = 19$ ($P < 0.00001$); $I^2 = 70\%$

Test for overall effect: $Z = 3.80$ ($P = 0.0001$)

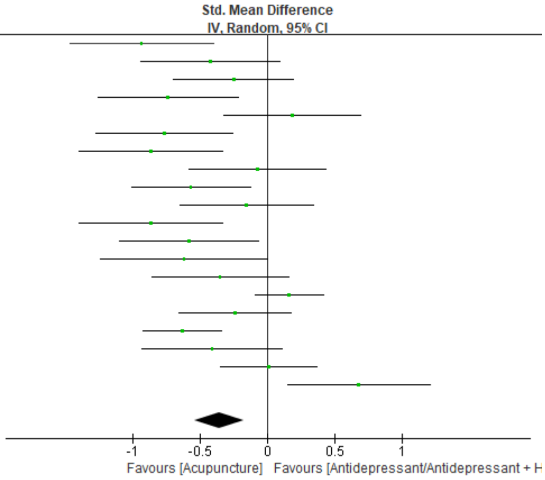

Appendix 9. Figures of meta-regression

Supplemental Figure 1. Univariate meta-regression based on publication year as covariate

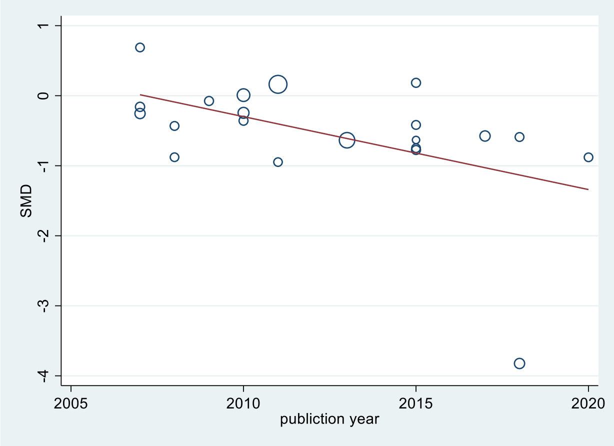

Supplemental Figure 2. Univariate meta-regression based on study sample size as covariate

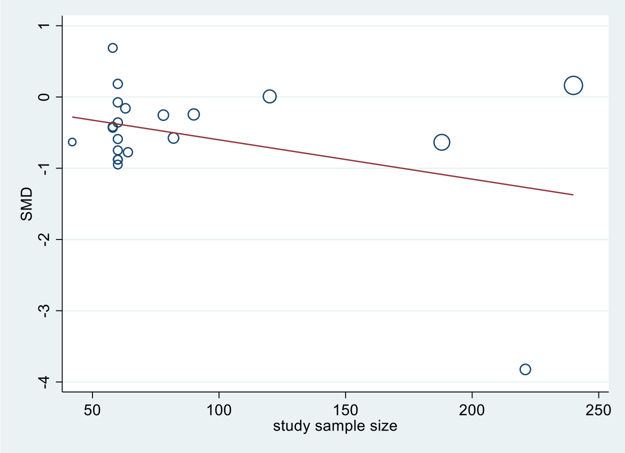

Supplemental Figure 3. Univariate meta-regression based on acupuncture stimulation as covariate

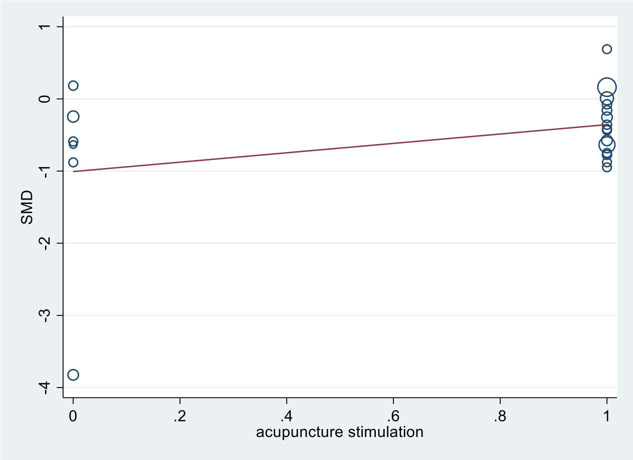

Supplemental Figure 4. Univariate meta-regression based on standard care in control groups as covariate

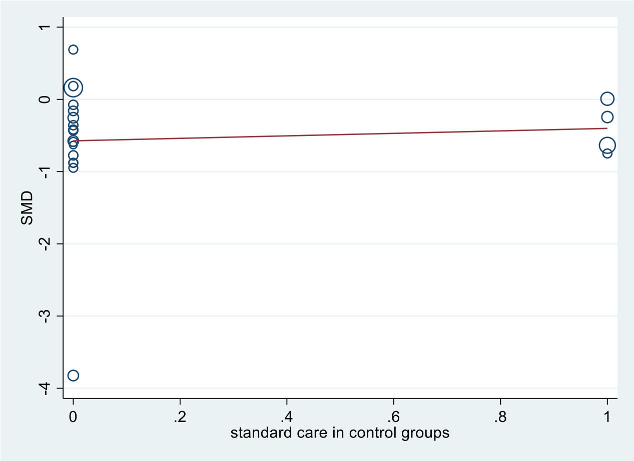

Supplement: Supplementary file 1 [file Data_Sheet_1.pdf]
